# Tumor microenvironment-targeted nanoparticles loaded with bortezomib and ROCK inhibitor improve efficacy in multiple myeloma

**DOI:** 10.1038/s41467-020-19932-1

**Published:** 2020-11-27

**Authors:** Cinzia Federico, Kinan Alhallak, Jennifer Sun, Kathleen Duncan, Feda Azab, Gail P. Sudlow, Pilar de la Puente, Barbara Muz, Vaishali Kapoor, Luna Zhang, Fangzheng Yuan, Matea Markovic, Joseph Kotsybar, Katherine Wasden, Nicole Guenthner, Shannon Gurley, Justin King, Daniel Kohnen, Noha N. Salama, Dinesh Thotala, Dennis E. Hallahan, Ravi Vij, John F. DiPersio, Samuel Achilefu, Abdel Kareem Azab

**Affiliations:** 1 Department of Radiation Oncology, Washington University School of Medicine, St. Louis, MO USA; 2 Department of Biomedical Engineering, Washington University, St. Louis, MO USA; 3 Department of Radiology, Washington University School of Medicine, St. Louis, MO USA; 4 Department of Pharmaceutical and Administrative Sciences, St. Louis College of Pharmacy, St. Louis, MO USA; 5 Department of Medicine, Washington University School of Medicine, St. Louis, MO USA; 6 Department of Pharmaceutics and Industrial Pharmacy, Faculty of Pharmacy, Cairo University, Cairo, Egypt

**Keywords:** Drug delivery, Nanoparticles, Cancer microenvironment, Myeloma

## Abstract

Drug resistance and dose-limiting toxicities are significant barriers for treatment of multiple myeloma (MM). Bone marrow microenvironment (BMME) plays a major role in drug resistance in MM. Drug delivery with targeted nanoparticles have been shown to improve specificity and efficacy and reduce toxicity. We aim to improve treatments for MM by (1) using nanoparticle delivery to enhance efficacy and reduce toxicity; (2) targeting the tumor-associated endothelium for specific delivery of the cargo to the tumor area, and (3) synchronizing the delivery of chemotherapy (bortezomib; BTZ) and BMME-disrupting agents (ROCK inhibitor) to overcome BMME-induced drug resistance. We find that targeting the BMME with P-selectin glycoprotein ligand-1 (PSGL-1)-targeted BTZ and ROCK inhibitor-loaded liposomes is more effective than free drugs, non-targeted liposomes, and single-agent controls and reduces severe BTZ-associated side effects. These results support the use of PSGL-1-targeted multi-drug and even non-targeted liposomal BTZ formulations for the enhancement of patient outcome in MM.

## Abstract (web summary)

The tumour microenvironment (TME) has a major role in chemoresistance in multiple myeloma. The authors show that a nanoparticle targeted to TME and loaded with bortezomib (BTZ) and Y27632 is more effective than free drugs, non-targeted and single-agent controls and reduces BTZ-related side effects.

## Introduction

Multiple myeloma (MM), the second most common hematological malignancy^1^, is characterized by the neoplastic transformation and growth of plasma cells within the bone marrow (BM). The advent of several therapeutic agents, including proteasome inhibitors (PIs) and immunomodulatory agents (IMiDs), has significantly improved the outcomes of MM treatment. However, almost all MM patients become refractory to treatment and relapse due to de novo drug resistance^2–4^.

We have previously studied the role of the BM microenvironment (BMME) in the development of drug resistance in MM. We found that direct interaction of MM cells with the BM stroma, endothelial cells (ECs), and extracellular matrix, as well as the cytokines and chemokines present in the BM milieu, induced drug resistance in MM cells^5–8^. Disruption of the interaction between MM cells and the BMME via inhibition of CXCR4 or selectins sensitized MM to therapy in vitro and in vivo^7–9^. The interaction of MM with stromal and ECs in the BMME is promoted through a signaling cascade that involves Rho guanosine triphosphatases and inhibition of their downstream targets, such as Rho kinase (ROCK), results in the abrogation of the MM–BMME interaction^5^. The combined use of chemotherapeutic and BMME-disrupting agents, such as bortezomib (BTZ) and a ROCK inhibitor, respectively, is an unexplored treatment approach for MM.

Serious side effects are the primary limitation for effective use of chemotherapies such as PIs and IMiDs in MM. Treatment with PIs is limited by their neurotoxicity, especially in the peripheral nerves, which leads to sensory axonal neuropathy^10,11^. Therefore, MM treatment strategies that specifically target MM cells, increase treatment efficacy, and reduce off-tumor side effects are urgently needed.

Recent efforts have focused on shifting from cytotoxic and non-specific chemotherapies to molecularly targeted and rationally-designed therapies that exhibit greater efficacy and fewer side effects^12^. Several studies have used nanoparticles for treatment of MM; however, most of these treatments were non-targeted, which led to considerable pharmacokinetic and pharmacodynamic disadvantages, including lack of specificity and dependency on the enhanced permeability and retention (EPR) effect^13^. We have recently shown that delivery of a PI, such as BTZ, in a CD38-targeted cross-linked chitosan nanoparticle, significantly reduced the toxicity profile of BTZ in vivo^14^. The anti-CD38 chitosan nanoparticles facilitated endocytosis-mediated uptake of CD38, which enhanced BTZ’s proteasome-inhibitory activity and specificity and resulted in a low toxicity profile to represent a promising therapy for MM. Despite this effect, the tumors eventually relapsed, most likely due to BMME-induced drug resistance.

In this study, we develop a P-selectin glycoprotein ligand-1 (PSGL-1)-decorated liposomes, targeting tumor-associated ECs, loaded with BTZ and BMME-disrupting ROCK inhibitor Y27632 to promote therapeutic efficacy and overcome TME-induced resistance. We find that targeting the BMME with PSGL-1-targeted BTZ and ROCK inhibitor-loaded liposomes is more effective than free drugs, non-targeted liposomes, and single-agent controls, and reduces BTZ-associated side effects, for the treatment of MM.

## Results

### Expression of P-selectin on MM-associated ECs in humans, in vivo, and in vitro

**Fig. 1:**
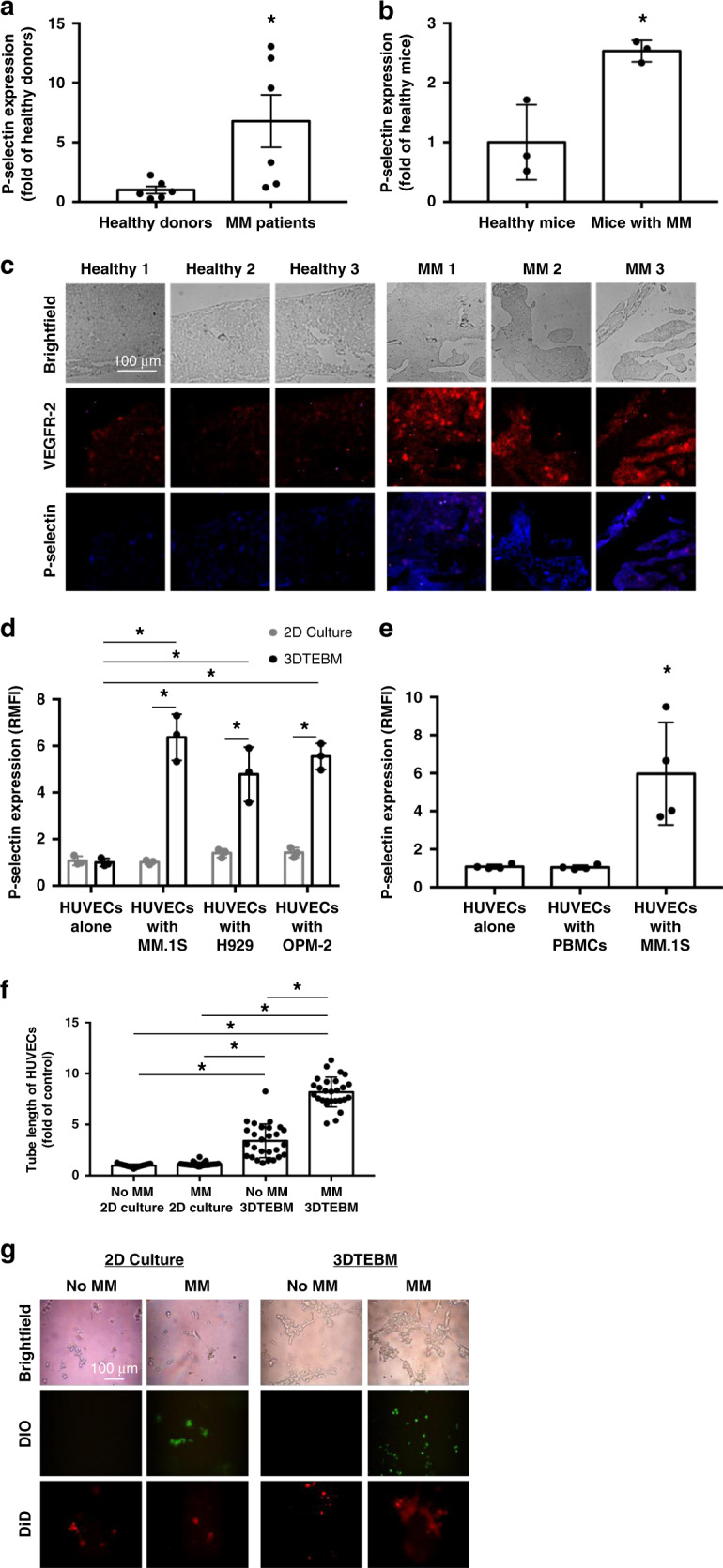
P-selectin is overexpressed on MM-associated endothelium. **a** P-selectin expression on endothelial cells (ECs) of healthy and (multiple myeloma) MM subjects (*p* value = 0.026; *n* = 6 samples examined over one independent experiment). Data presented here are defined as mean values +/− SEM. **b** Expression of P-selectin on the BM endothelium of healthy and MM-inoculated mice (*p* value < 0.001; *n* = 3 samples examined over one independent experiment). **c** Immunofluorescence of VEGFR-2 and P-selectin on sections from BM of healthy and MM-inoculated mice. **d** P-selectin expression in ECs cultured alone or with MM cells in 2D culture or the 3DTEBM (all *p* values shown are <0.01; *n* = 3 samples examined over one independent experiment). **e** P-selectin expression of ECs when incubated alone, with PBMCs, or MM (*p* value = 0.01; *n* = 3 samples examined over one independent experiment). **f** Quantitative analysis and **g** qualitative images of tube-like formation on Matrigel (2D Culture) and the 3DTEBM with and without MM cells (all *p* values shown are < 0.01; *n* = 27 cells examined over one independent experiment). Experiments seen here were repeated three times independently. All data presented here are defined as mean values +/− SD unless otherwise noted. Statistical significance was analyzed using an unpaired Student’s two-sided *t*-test (*p < 0.05).

Using flow cytometry, we investigated the expression of P-selectin in healthy and tumor-associated ECs, in primary healthy and MM patient BM samples, respectively. The gating strategy is shown in Supplementary Fig. 1. We demonstrated that expression of P-selectin was 6-fold higher on ECs from the BM of patients who had newly diagnosed or relapsed MM compared to ECs from the BM of healthy donors (Fig. 1a). Similarly, we tested the expression of P-selectin in mice in ECs isolated from BM of healthy and MM-bearing, using flow cytometry. We found a 2.5-fold higher increase of P-selectin expression on ECs from the BM of MM-bearing mice compared to ECs from healthy mice (Fig. 1b). We also investigated the expression of P-selectin on ECs (VEGFR-2+ cells) in the femurs of healthy and MM-bearing mice via immunofluorescence. The presence and level of P-selectin expression co-localized with VEGFR-2+ ECs was greater on ECs in the BM of MM-bearing mice compared with healthy mice (Fig. 1c).

To better simulate the BM niche, we developed the 3D tissue-engineered bone marrow (3DTEBM) using BM plasma derived from MM patients which is shown in Supplementary Fig. 2a^15^. Human umbilical vein ECs (HUVECs, cyan) were plated on top of the 3DTEBM; stromal (red) and MM (green) cells were plated inside the 3DTEBM matrix (Supplementary Fig. 2b). The MM cells were dispersed throughout the scaffold whereas the stromal cells coalesced toward the bottom of the culture, biomimicking the BM niche.

Next, we examined the expression of P-selectin in HUVECs cultured alone or with MM cells in traditional 2D culture (Matrigel) or in the 3DTEBM. In the 3DTEBM, we observed a 6-fold increase in P-selectin expression on the HUVECs cultured with MM (MM.1S, H929, or OPM-2) compared to ECs alone (Fig. 1d). In traditional 2D cultures, P-selectin expression did not significantly increase even when co-cultured with MM cell lines. We also investigated whether or not P-selectin expression increased when co-cultured with non-malignant cells. In Fig. 1e, we found that, unlike the co-culture of ECs with MM, co-culture of ECs and PBMCs did not increase in P-selectin expression in ECs. In addition, HUVECs were plated on Matrigel alone (2D) or on top of the 3DTEBM with and without MM. We saw that the ECs formed significantly more tube-like features when co-cultured with MM in the 3DTEBM, compared to ECs alone without MM cells or in 2D conditions (Fig. 1f and g).

### Binding of PSGL-1-targeted liposomes to P-selectin and MM-associated ECs in vitro and in vivo

**Fig. 2:**
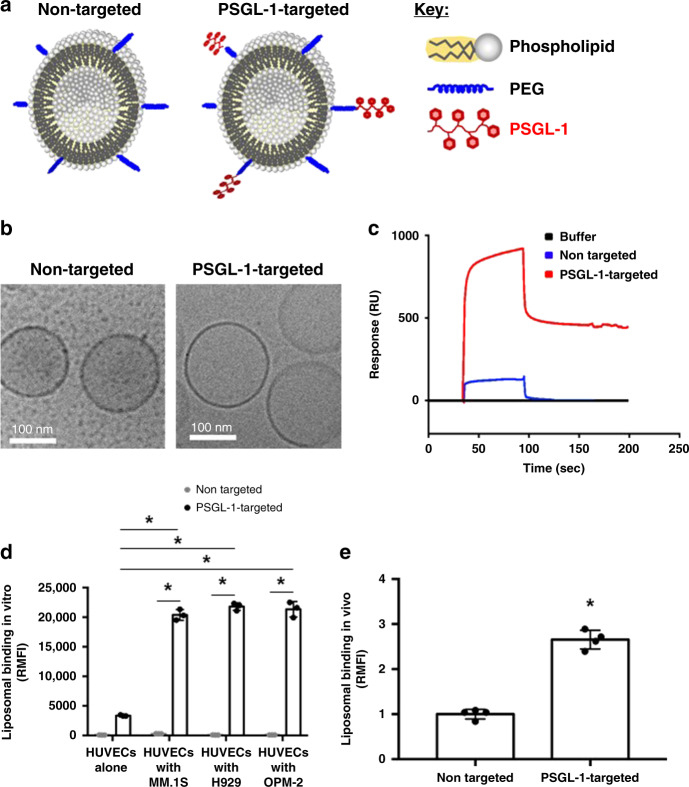
PSGL-1 specifically binds to P-selectin. **a** Illustrations of non-targeted and P-selectin glycoprotein ligand-1 (PSGL-1)-targeted liposomes. **b** Cryogenic transmission electron microscopy images of non-targeted and PSGL-1-targeted liposomes. **c** The binding rate of PSGL-1 to P-selectin assessed using BIAcore. **d** Liposomal binding of PSGL-1-targeted and non-targeted particles to ECs in vitro (all *p* values shown are < 0.001; *n* = 3 samples examined over one independent experiment). **e** Liposomal binding of PSGL-1-targeted and non-targeted particles to ECs in vivo (*p* value < 0.001; *n* = 4 samples examined over one independent experiment). Experiments seen here were repeated three times independently. All data presented here are defined as mean values +/− SD. Statistical significance was analyzed using an unpaired Student’s two-sided *t*-test (*p < 0.05).

Next, we developed PSGL-1-targeted liposomes to target the overexpressed P-selectin on MM-associated endothelium; a schematic of these liposomes is shown in Fig. 2a. The physical characteristics of the PSGL-1-targeted and non-targeted liposomes are summarized in Supplementary Table 1 and cryogenic transmission electron microscopy (cryoTEM) images are shown in Fig. 2b. To evaluate the affinity of PSGL-1-targeted liposomes for P-selectin, we assessed the binding of the liposomes to P-selectin. The successful immobilization of P-selectin to the surface plasmon resonance sensor chip is shown in Supplementary Fig. 3. We found that, compared to non-targeted, PSGL-1-targeted liposomes showed an 8-fold increase in binding to recombinant P-selectin (Fig. 2c). We also tested the binding of the PSGL-1- and non-targeted liposomes to naive and tumor-associated ECs in the 3DTEBM described in Fig. 1. We found that the non-targeted liposomes exhibited negligible binding to the endothelium; while the PSGL-1-targeted liposomes had significantly higher binding (7-fold) to the tumor-associated endothelium compared to the naive endothelium (Fig. 2d). In addition, we tested the binding of the PSGL-1-targeted and non-targeted liposomes to tumor-associated ECs in the BM of MM-bearing mice. We found that PSGL-1-targeted liposomes exhibited significantly greater binding to the MM-associated endothelium compared to non-targeted liposomes (Fig. 2e).

### Targeting the tumor microenvironment with PSGL-1 in vivo

**Fig. 3:**
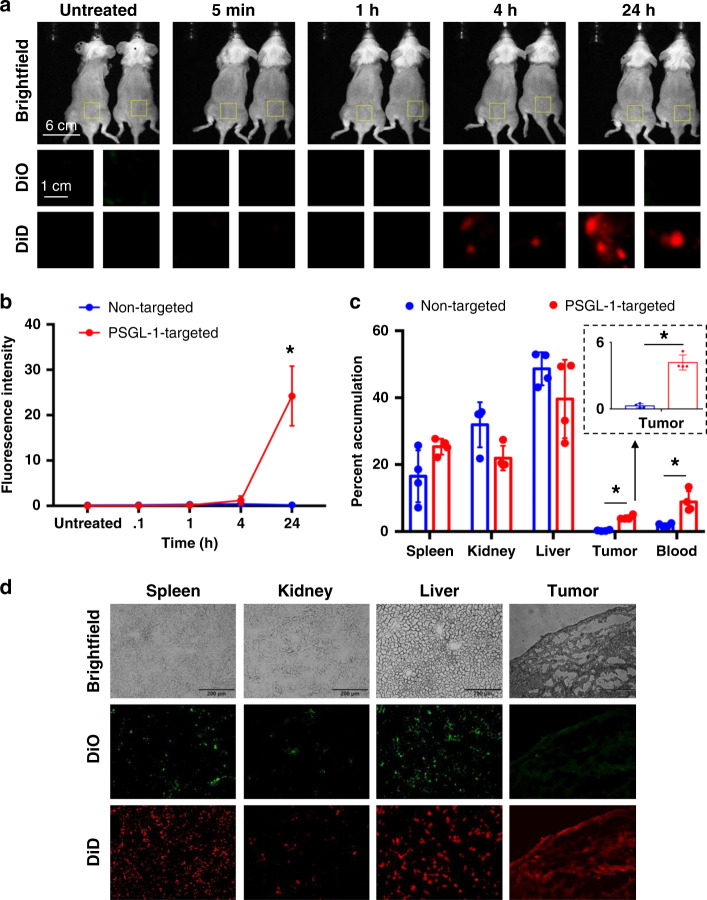
Targeting the tumor microenvironment with PSGL-1 in vivo. **a** Tumor accumulation of non-targeted (DiO) and PSGL-1-targeted (DiD) liposomes at *t* = 5 min, 1, 4, and 24 h. **b** Quantitative assessments of tumor accumulation in vivo (*p* value < 0.01). **c** Percent accumulation of non-targeted and PSGL-1-targeted liposomes in extracted tissues (spleen, kidney, liver, blood, and tumor) post-mortem 24 h post-injection (all *p* values shown are < 0.01). **d** Histology images of the extracted tissues 24 h post-injection for non-targeted (DiO) and PSGL-1-targeted (DiD) liposomes. For the experiments seen here, each mouse represents an independent replicate. All data presented here are defined as mean values +/− SD. Statistical significance was analyzed using an unpaired Student’s two-sided *t*-test or one-way analysis of variance (*p < 0.05).

Following the successful targeting of the tumor microenvironment in Fig. 2e, we subsequently injected MM.1S subcutaneously in NCG mice. Non-targeted and PSGL-1-targeted liposomes colored with DiO and DiD, respectively, were mixed and injected intravenously following tumor propagation. As time progressed, we observed a higher accumulation of the PSGL-1-targeted liposomes in the tumor area compared to non-targeted after 24 h (Fig. 3a). There is a significantly higher presence of PSGL-1-targeted liposomes after 24 h as shown quantitatively in Fig. 3b. Following 24 h, we extracted the organs of these mice and assessed the percent accumulation in each organ 24 h post-injection (Fig. 3c). As expected, we found significant accumulation in the spleen, kidney, and liver for both types of liposomes. A significantly higher presence of PSGL-1-liposomes was detected in the blood and tumor compared to non-targeted. We then assessed the accumulation of liposomes using fluorescence microscopy of  histology sections of the tissues which further confirmed the preferential accumulation of the PSGL-1-targeted liposomes in the tumor site compared to non-targeted (Fig. 3d).

### Encapsulation of BTZ and Y27632 in liposomes

**Fig. 4:**
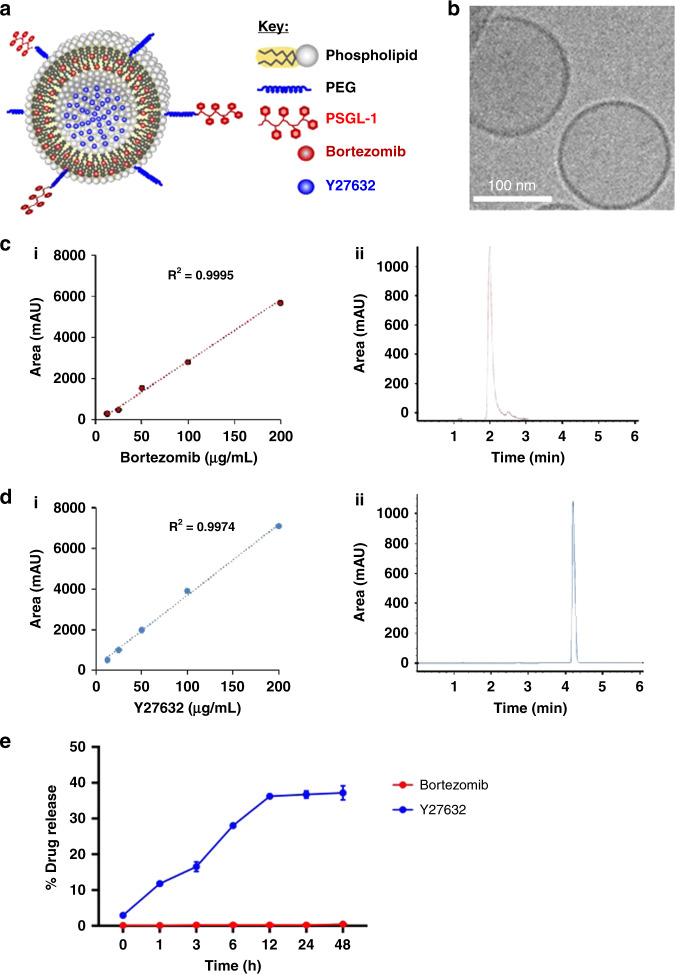
Loading of BTZ and Y27632 into liposomes. **a** Schematic of PSGL-1 combination (BTZ and Y27632) nanoparticulate therapy. **b** Cryogenic transmission electron microscopy images of BTZ and Y27632-encapsulated liposomes. **c** (i) HPLC calibration curve for BTZ. (ii) HPLC detection peak for BTZ. **d** (i) HPLC calibration curve for Y27632. (ii) HPLC detection peak for Y27632. **e** Drug release of BTZ and Y27632 over a 48-h period. Experiments seen here were repeated three times independently.

We loaded the therapeutic (BTZ) and BMME-disrupting agents (Y27632) into the lipid bilayer and hydrophilic core of the liposomes, respectively (Fig. 4a), and the physical characterization of the loaded liposomes are shown in Supplementary Table 2. CryoTEM images of the loaded liposomes are seen in Fig. 4b. We developed high-performance liquid chromatography (HPLC)-analysis methods for both BTZ and Y27632 (Fig. 4c and d). We found that BTZ and Y27632 had retention times of 2.1 and 4.1 min, respectively, with linear calibration curves in the range of 12.5–200 μg/ml and a linear correlation coefficient of 0.99. We then evaluated the encapsulation efficiency (EE) of the liposomes, and found that the maximal EEs for BTZ and Y27632 were 77% and 55%, respectively. In addition, we evaluated the drug release of the encapsulated liposomes over time and found negligible release of BTZ and 40% release of Y27632 over 48 h (Fig. 4e).

### Effect of Y27632-loaded liposomes on adhesion signaling, MM trans-endothelial migration in vitro, and MM extravasation in vivo

**Fig. 5:**
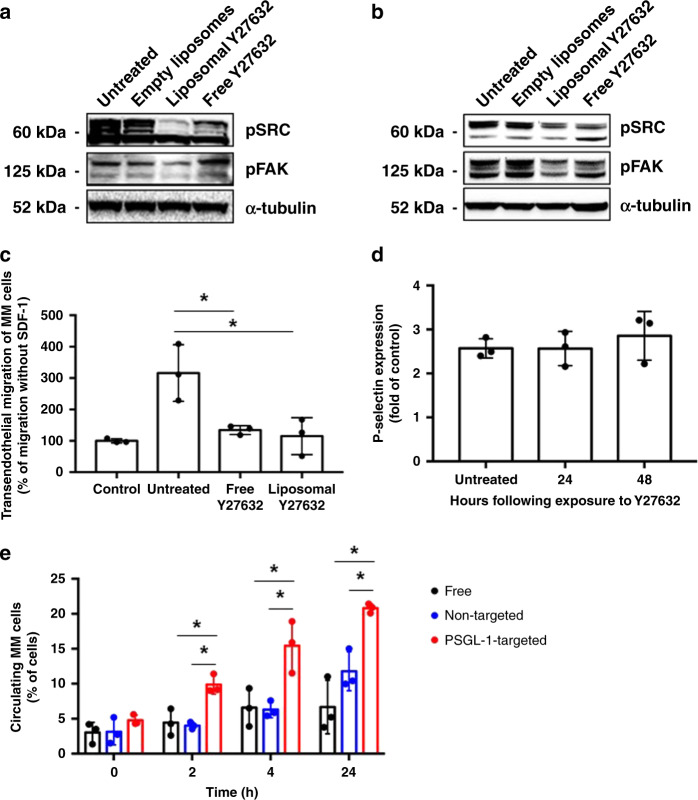
Y27632 reduces adhesion in MM and ECs in vitro and in vivo. **a** FAK and SRC signaling in MM following liposomal or free Y27632 treatment. **b** FAK and SRC signaling in ECs following liposomal or free Y27632 treatment. **c** Trans-endothelial migration of MM cells with and without the chemokine SDF-1 with free and liposomal Y27632 in vitro (all *p* values shown are <0.05; *n* = 3 samples examined over one independent experiment). **d** Effect of P-selectin with exposure of Y27632 (*n* = 3 samples examined over one independent experiment). **e** Percentage of MM cells circulating in the peripheral blood following treatment with free, non-targeted, or PSGL-1-targeted Y27632 in vivo (all *p* values shown are <0.05; *n* = 3 samples examined over one independent experiment). Experiments seen here were repeated three times independently. All data presented here are defined as mean values +/− SD. Statistical significance was analyzed using an unpaired Student’s two-sided *t*-test (*p < 0.05).

We compared the effect of free and liposomal Y27632 on adhesion signaling in MM cells and HUVECs. We found that empty liposomes did not have an effect on adhesion signaling when compared to untreated, while free Y27632 decreased adhesion signaling as previously shown^5^. Liposomal Y27632 decreased adhesion (pSRC and pFAK) similarly to free Y27632 in ECs. Interestingly, liposomal Y27632 decreased pFAK and pSRC expression compared to untreated, empty liposomes, and free Y27632 in MM cells (Fig. 5a, b). Quantitative assessments of the data in Fig. 5a, b are shown in Supplementary Fig. 4a-d.

Functionally, both free and liposomal Y27632 reversed stromal cell-derived factor (SDF)-induced trans-endothelial migration of MM cells in vitro (Fig. 5c). Furthermore, the expression of P-selectin on HUVECs did not change with the exposure of Y27632 following a duration of 48 h (Fig. 5d). In vivo, non-targeted Y27632-loaded liposomes resulted in mobilization of MM cells to the blood circulation at 2, 4, and 24 h in a similar manner to free drug. PSGL-1-targeted Y27632-loaded liposomes induced greater mobilization of MM cells to the blood circulation at 2, 4, and 24 h, reflecting a greater inhibition of the MM–BMME interaction than that of the free and non-targeted forms of Y27632 due to the ability of targeting P-selectin (Fig. 5e).

### Effect of BTZ-loaded liposomes on survival, proliferation, and apoptosis signaling in MM and HUVECs in vitro

**Fig. 6:**
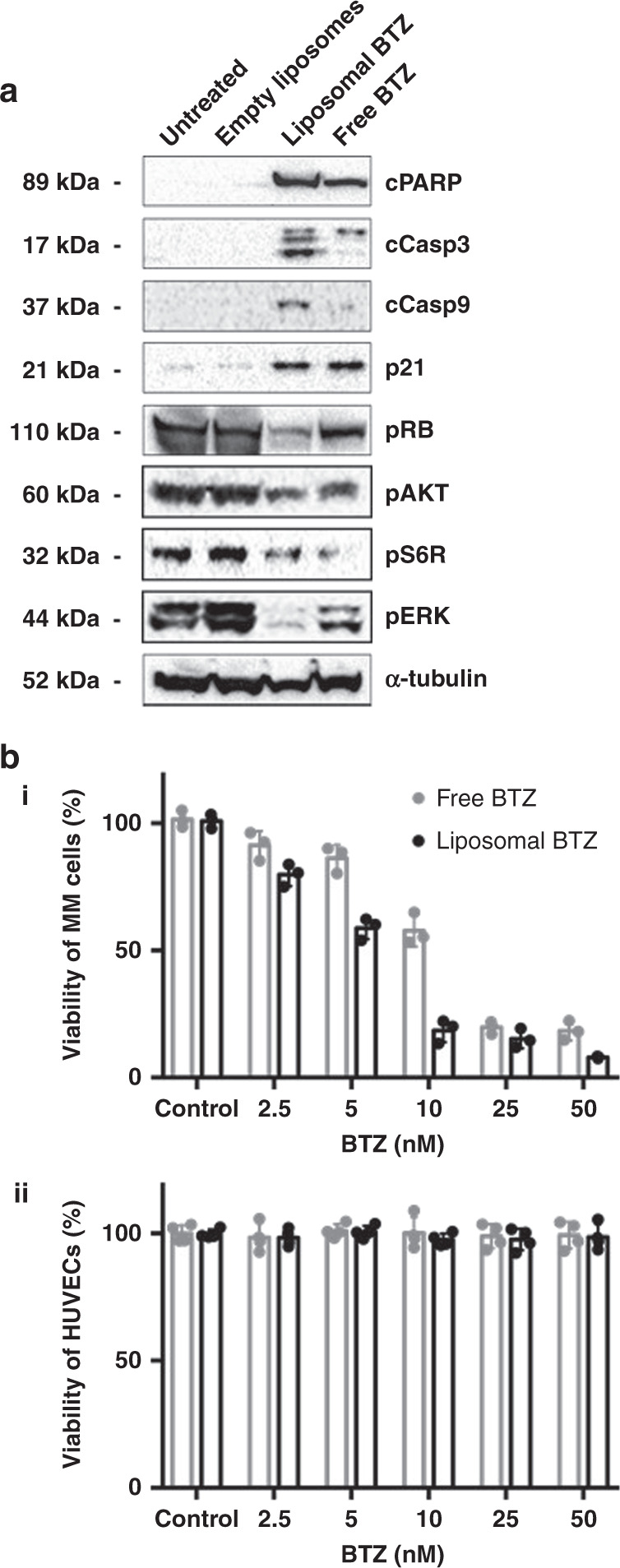
BTZ induces apoptosis only in MM. **a** Immunoblotting of factors related to apoptosis (cPARP, p21, cCasp3, and cCasp9), cell cycle (pRB), and survival (pAKT, pS6R, and pERK) following treatment with empty liposomes, liposomal BTZ, or free BTZ. **b** (i) Viability of MM cells following a 24-h incubation with increasing concentrations of BTZ. (ii) Viability of ECs following a 24-h incubation with increasing concentrations of BTZ. Experiments seen here were repeated three times independently. All data presented here are defined as mean values +/− SD.

We compared the effect of free and liposomal BTZ on apoptosis and proliferation signaling in MM cells. While empty liposomes did not have any effect on apoptosis and proliferation signaling, we found that liposomal BTZ increased pro-apoptotic signaling (cPARP, cCasp3, and cCasp9) and decreased proliferation (pRb, pAKT, pS6R, and pERK) more profoundly compared to free BTZ (Fig. 6a). Functionally, we found that liposomal BTZ killing of MM cells was more effective than that of free BTZ in vitro, with half-maximal inhibitory concentration (IC50) values of ~5 and 10 nM, respectively (Fig. 6bi). BTZ, in addition, did not affect the survival of HUVECs (Fig. 6bii).

### Effect of PSGL-1-targeted, BTZ- and Y27632-loaded liposomes on MM tumor progression in vivo

To test the efficacy of the PSGL-1-targeted, BTZ- and Y27632-loaded liposomes on MM tumor progression, we utilized 12 treatment groups of MM-bearing mice: (i) vehicle, (ii) BTZ as a free drug, (iii) Y27632 as a free drug, (iv) combination of BTZ and Y27632 as free drugs, (v) non-targeted empty liposomes, vi) non-targeted BTZ-loaded liposomes, (vii) non-targeted Y27632-loaded liposomes, (viii) non-targeted multi-drug (BTZ and Y27632) liposomes, (ix) PSGL-1-targeted empty liposomes, (x) PSGL-1-targeted BTZ-loaded liposomes, (xi) PSGL-1-targeted Y27632-loaded liposomes, and (xii) PSGL-1-targeted multi-drug (BTZ and Y27632) liposomes.

**Fig. 7:**
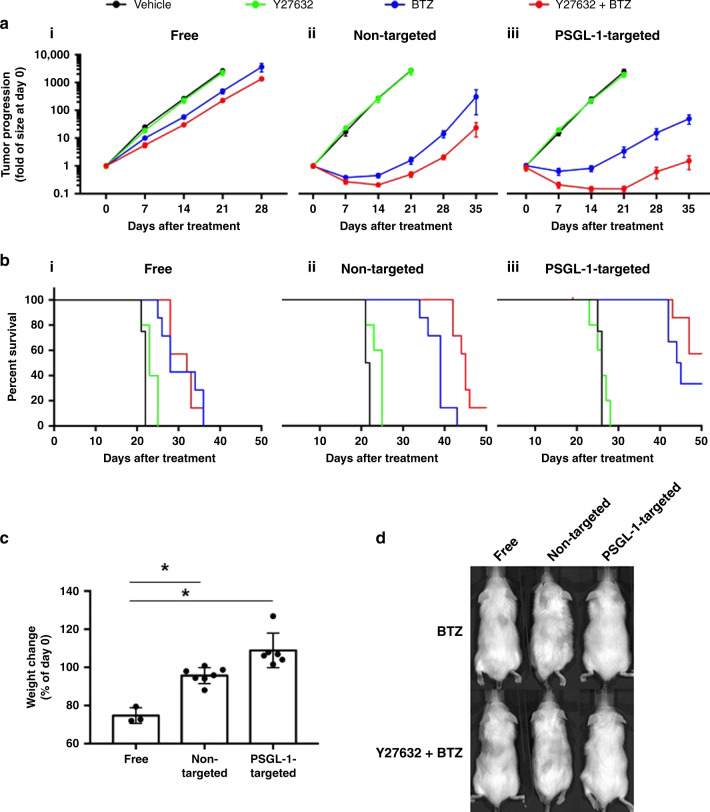
PSGL-1 combination nanoparticle therapy decreased tumor burden, prolonged survival, and reduced adverse events in vivo. **a** MM burden of mice treated with (i) free, (ii) non-targeted, and (iii) PSGL-1-targeted forms of treatment (vehicle, Y27632, BTZ, and Y27632 + BTZ) measured using bioluminescence. Data presented here are defined as mean values +/−SEM. **b** Survival of mice treated with (i) free, (ii) non-targeted, and (iii) PSGL-1-targeted drugs. **c** Percent weight change of mice in each group; each group was stratified by the form of treatment injected (all *p* values shown are <0.05; *n* = 3, *n* = 7, and *n* = 6 samples examined over one independent experiment for the groups: free, non-targeted, and PSGL-1-targeted, respectively). Data presented here are defined as mean values +/−SD. **d** Representative images of hair loss experienced in vivo following BTZ and combination treatments (free, non-targeted, and PSGL-1-targeted). For the experiments seen here, each mouse represents an independent replicate. Statistical significance was analyzed using an unpaired Student’s two-sided *t*-test (*p < 0.05).

We found that the groups treated with non-targeted empty liposomes and PSGL-1-targeted empty liposomes displayed tumor growth rates similar to that of the vehicle-treated control (Supplementary Fig. 5a). Y27632 as a free drug, in non-targeted liposomes, or in PSGL-1-targeted liposomes also did not affect tumor growth (Supplementary Fig. 5b). However, free BTZ significantly delayed tumor growth, and combining BTZ with Y27632 only slightly enhanced this effect (Fig. 7ai); statistical analyses of tumor efficacy in Fig. 7 are shown in Supplementary Table 3. Non-targeted BTZ-loaded liposomes significantly reduced tumor progression by approximately three orders of magnitude when compared to non-targeted empty liposomes. The non-targeted multi-drug (BTZ and Y27632) liposomes were significantly more effective than BTZ-loaded liposomes (Fig. 7aii). Treatment with PSGL-1-targeted BTZ-loaded liposomes resulted in a significant reduction in tumor progression (approximately three orders of magnitude) compared to non-targeted empty liposome controls; PSGL-1-targeted multi-drug liposomes significantly improved the antitumor effect and reduced tumor progression by an order of magnitude compared to the PSGL-1-targeted BTZ-loaded liposomes (Fig. 7aiii). Compared to free BTZ, the non-targeted and PSGL-1-targeted liposomal BTZ formulations resulted in a significant decrease in tumor progression (Supplementary Fig. 5c); statistical analyses of Supplementary Fig. 4 are shown in Supplementary Table 4. The combination of BTZ and Y27632 was more effective than BTZ alone in only the liposomal formulations (non-targeted and PSGL-1-targeted). The group treated with PSGL-1-targeted multi-drug liposomes displayed the greatest reduction in tumor progression 35 days after treatment when compared to the other 11 treatment groups (Supplementary Fig. 5d).

In addition to tumor progression, we also assessed the survival of MM-bearing mice treated with the various treatments described above. We found that the mice treated with non-targeted empty liposomes and PSGL-1-targeted empty liposomes died around the same time as the vehicle-treated controls (Supplementary Fig. 6a). In addition, Y27632 alone as a free drug, in non-targeted liposomes, or in PSGL-1-targeted liposomes did not extend the survival of the MM-bearing mice (Supplementary Fig. 6b). Free BTZ significantly prolonged the survival of the mice compared to untreated; however, this effect was not improved when BTZ was combined with Y27632 (Fig. 7bi); statistical analyses of survival curves in Fig. 7 are shown in Supplementary Table 3. Mice treated with non-targeted BTZ-loaded liposomes lived significantly longer than mice treated with vehicle or Y27632-loaded liposomes, and non-targeted multi-drug liposomes significantly improved the rate of survival past 50 days from 0% (BTZ-only liposomes) to 14% (Fig. 7bii). PSGL-1-targeted BTZ-loaded liposomes significantly extended the survival of the mice when compared to the PSGL-1-targeted empty and Y27632-loaded liposomes, and PSGL-1-targeted multi-drug liposomes slightly increased the survival rate from ~30% (PSGL-1-targeted BTZ-only liposomes) to 60% (Fig. 7biii). Non-targeted BTZ-loaded liposomes extended the survival of MM-bearing mice, an effect that was significantly enhanced when treated with PSGL-1-targeted BTZ liposomes (Supplementary Fig. 6c); statistical analyses of Supplementary Figs. 5 and 6 are shown in Supplementary Table 4. The combination of BTZ and Y27632 prolonged survival in the non-targeted and PSGL-1-targeted formulations compared to free BTZ and Y27632 (Supplementary Fig. 6d). Moreover, targeting P-selectin with PSGL-1-targeted liposomes prolonged the survival of BTZ and multi-drug groups of mice.

### Effect of PSGL-1-targeted, BTZ-, and Y27632-loaded liposomes on BTZ-associated side effects in vivo

In parallel with the above experiments, we also assessed the occurrence and severity of BTZ-associated side effects in the treatment groups. As expected, the free-drug treatments induced significant weight loss, whereas the non-targeted and PSGL-1-targeted treatments resulted in significantly reduced weight loss compared to the free-drug regimen. Notably, the weights of the mice treated with the PSGL-1-targeted treatments actually increased by ~5% compared to their weights measured prior to therapy (Fig. 7c). The non-targeted forms of treatment decreased hair loss compared to the free drug, and the use of PSGL-1-targeted liposomes resulted in absolutely no hair loss (Fig. 7d).

## Discussion

Drug resistance and dose-limiting toxicities remain significant barriers for the treatment of MM and cancer in general. The BMME directly interacts with MM cells and induces pleiotropic signaling that confers tumorigenesis and drug resistance in MM cells^16^. BTZ is the first FDA-approved PI and one of the frontline regimens used for the treatment of MM^17^. Despite the clinical success of BTZ in the past two decades, its dose-limiting toxicities and the development of drug resistance hinder its ability to fully eradicate MM^18^. Thus, approaches to improve the efficacy and reduce the toxicity of promising treatments such as BTZ are urgently needed.

One potential approach to improve the efficacy and reduce the toxicity of treatment is to encapsulate the PI in a nanoparticle and add targeting elements which will increase the specific accumulation of the particle (and the PI within) to the tumor^13,19^. We previously demonstrated that BTZ loaded into a chitosan nanoparticle and decorated with anti-CD38 antibodies improved the accumulation of BTZ in MM cells, which overexpress CD38, and reduced the toxicity of BTZ in normal tissue^14^. However, several studies have shown that the first tissue barrier nanoparticles face are ECs in the blood vessels adjacent to the tumor rather than the tumor cells themselves^13,19^. Therefore, in this study, we aimed to target the tumor-associated endothelium as a unique target in the tumor area.

We previously demonstrated that PSGL-1 (the natural ligand of P-selectin) plays a critical role in the interaction of MM cells with ECs and is involved in MM cell adhesion and homing to the BM^7,9^. Therefore, in this study, we hypothesized that the receptor of PSGL-1 (P-selectin) is highly and specifically expressed on the endothelium in the vicinity of MM cells, which can be used as a unique target for specific drug delivery to the tumor area. We found that P-selectin expression was highly upregulated on MM-associated endothelium compared to healthy endothelium in MM patients, MM-bearing mice, and 3DTEBM cultures in vitro (Fig. 1). Notably, P-selectin overexpression was not observed in classic 2D co-cultures of MM cells and ECs, but was overexpressed in ECs in the 3DTEBM, a model which we further utilized for binding assays. We found that PSGL-1-targeted liposomes specifically bound to MM-associated endothelium compared to normal endothelium in vitro and in vivo (Figs. 2 and 3). These results agree with previous observations of P-selectin overexpression in tumor-associated endothelium in glioblastoma, lung, ovarian, lymphoma, breast, and other cancer subtypes^20,21^, which suggests that PSGL-1 could be used to target the tumor-associated endothelium in other cancer subtypes.

The limitations of cancer therapy not only include lack of specificity but also the development of drug resistance over time. For instance, we previously demonstrated that the interaction between MM cells and their BMME plays a crucial role in the development of resistance to therapy^5–8^. We demonstrated that using a BMME-disrupting agent, such as the CXCR4 inhibitor AMD3100, re-sensitized MM cells to BTZ in vivo^8^. The combination treatment containing AMD3100 (Plerixafor) and BTZ was also translated to a clinical trial (NCT00903968) where an encouraging 48.5% overall response rate in relapsed or refractory MM patients was observed^22^. However, following completion of this trial, multiple issues remained that hinder a physician’s ability to routinely prescribe Plerixafor to MM patients. First, Plerixafor has a pharmacokinetic (PK) half-life between 3 and 5 h^23^, which severely hinders efficient drug administration, as the drug needed to be infused for 6 consecutive days, resulting in significant discomfort to the participating patients. Second, the PK half-life of Plerixafor is significantly shorter than the half-life of BTZ [40 h^24^], which hinders determination of an effective combinatorial and synchronized treatment schedule. Third, the Plerixafor and BTZ combination treatment induced various adverse side effects, such as sensory neuropathy, to the participants in the clinical study. Hence, we hypothesized that a nanoparticulate delivery system with dual loading of chemotherapy and a BMME-disrupting agent will overcome the mentioned PK issues and ensure simultaneous delivery of the two agents to the desired target.

To test this hypothesis, we combined BTZ with a BMME-disrupting agent, a ROCK inhibitor, into liposomes. We used the ROCK inhibitor in place of Plerixafor in this formulation because Plerixafor is a CXCR4 inhibitor and needs to be released in the extracellular milieu to inhibit the extracellular domain of CXCR4; while BTZ needs to be internalized into the cell to inhibit the proteasome. The two drugs’ different sites of action and release kinetics would present significant challenges in their dual delivery. Therefore, we paired BTZ with a ROCK inhibitor that acts as a BMME-disrupting agent and targets an intracellular kinase. We have previously shown that this compound inhibits the interaction between MM cells and their BMME, similar to Plerixafor^5^.

The ROCK inhibitor, Y27632, was loaded to the aqueous core of the liposomes, while BTZ was loaded to the lipid bilayer (Fig. 4). We found that liposomal Y27632 downregulated adhesion signaling (SRC and FAK) in MM and BMME cells in vitro, reduced trans-endothelial migration of MM cells in vitro, and increased mobilization of MM cells in vivo; the effect of liposomal Y27632 was similar to or greater than that of free Y27632 (Fig. 5). Similarly, we found that liposomal BTZ downregulated proliferation signaling, increased apoptosis signaling, and induced cytotoxicity in MM cells, but not in ECs in vitro, which is consistent with previously published findings^25^. On the other hand, there are published studies that have seen a decrease in viability and inhibition of cellular growth of ECs when treated with BTZ; however, these studies observe cell death when the concentration of BTZ reaches 100 nM to 1 μM^26^. In addition, the effect of liposomal BTZ was similar to or greater than that of free BTZ (Fig. 6).

We then assessed the efficacy of the targeted combination therapy of BTZ and Y27632 in MM-bearing mice in vivo. We found that combining a PI with a BMME-disrupting agent was a successful approach to increase MM sensitivity to therapy and overcome BMME-induced drug resistance in the liposomal forms of delivery (non-targeted liposomes and PSGL-1 targeted liposomes). In the PSGL-1-targeted formulation, the combination of BTZ with Y27632 resulted in a significantly better tumor efficacy and survival compared to all other treatment groups (Fig. 7).

Compared to administration as a free drug, delivery of BTZ with liposomes improved the therapeutic efficacy of BTZ alone or in combination with Y27632; this result is likely due to specific accumulation of the drugs in the tumor due to the EPR effect of liposomes^13^. However, the use of PSGL-1-targeted liposomes further increased the specificity and therapeutic efficacy of BTZ when combined with Y27632 due to their specific interaction with the MM-associated endothelium.

No difference was found between PSGL-1-targeted and non-targeted liposomal BTZ. We hypothesize that this is due to the fact that BTZ did not have a significant killing effect on ECs themselves (as shown in Fig. 6bii); therefore, the effect was mainly due to the passive (EPR effect) accumulation in the tumor, and further targeting to ECs will not create an additional effect. In contrast, the PSGL-1-targeted multi-drug combination had a better effect than the non-targeted multi-drug formulation (Supplementary Fig. 5d). We hypothesize that this is due to the direct effect of the ROCK inhibitor on the ECs, which in turn disrupted their interaction with MM cells and made the MM cells more sensitive to BTZ (which accumulated in the tumor area by passive EPR effect and/or by active targeting to the tumor-associated endothelium).

The combinatorial effect of BTZ and Y27632 was more dramatic in the liposomal formulations than as free drugs due to their synchronized delivery, and the effect was even more pronounced in the PSGL-1-targeted liposomes. Furthermore, PSGL-1-targeted liposomes reduced the side effects of BTZ; these liposomes did not cause weight or hair loss when compared to the non-targeted liposomes and free drugs (Fig. 7).

In conclusion, PSGL-1-targeted, BTZ- and Y27632-loaded liposomes, which target the MM-associated endothelium and synchronize the delivery of PI with BMME-disrupting agents, demonstrated improved specificity and efficacy and reduced the side effects when compared to free drugs and non-targeted liposomes. These results support the use of PSGL-1-targeted multi-drug and even non-targeted liposomal BTZ formulations for the enhancement of patient outcome in MM.

## Methods

### Materials and reagents

The phospholipids, 1,2-dipalmitoyl-sn-glycero-3-phosphocholine (DPPC), 1,2-distearoyl-sn-glycero-3-phosphoethanolamine-N-[methoxy(polyethylene glycol)-2000] (DSPE-mPEG2000), and 1,2-distearoyl-sn-glycero-3-phosphoethanolamine-N-[succinyl(polyethylene glycol)-2000] (DSPE-PEG(2000)-succinyl) were purchased from Avanti Polar Lipids, Inc. (Alabaster, AL). Cholesterol (Chol), N-(3-Dimethylaminopropyl)-N′-ethylcarbodiimide hydrochloride (EDC), and N-Hydroxysuccinimide (NHS) were purchased from Sigma-Aldrich (St. Louis, MO, USA). Calcein violet and lipophilic tracers (DiO and DiD) were purchased from Invitrogen (Eugene, OR). BTZ and Y27632 were purchased from MedKoo Biosciences (Morrisville, NC). Recombinant PSGL-1 protein was purchased from Novoprotein (Summit, NJ). Monoclonal antibodies (mAb) used for immunoblots were purchased from Cell Signaling Technology (Danvers, MA). Phospho-Akt (pAKT; #4060), phospho-Erk1/2 (pERK; #4370), phospho-Rb (pRB; #9308), p21 (#2947), cleaved Caspase3 (cCasp3; #9664), cleaved Caspase 9 (cCasp9; #7237), cleaved PARP (cPARP; #5625), phospho-FAK (pFAK; #3284), phospho-SRC (pSRC; #6943), and phospho-S6 ribosomal protein (pS6R; #4858) were all used at a dilution of 1:1000. α-Tubulin (#2125) was used as a loading control at a dilution of 1:3000. The immunoblots were detected using an ECL Plus chemiluminescent system (PerkinElmer, Waltham, MA). The mAbs used for flow cytometry were purchased from Miltenyi Biotec (Bergisch Gladbach, Germany) unless otherwise noted.

### Cell culture

The MM cell lines, MM.1S and H929, were purchased from the American Type Culture Collection (ATCC, Rockville, MD), OPM-2 and green fluorescent protein-labeled and luciferase-transfected MM.1S (MM.1S-GFP-Luc) were a kind gift from Dr. Irene Ghobrial (Dana-Farber Cancer Institute, Harvard Medical School, Boston, MA). Human umbilical vein endothelial cells (HUVECs) were purchased from Angio-Proteomie (Boston, MA). Human samples for this study were collected under informed consent, in concordance with Washington University Institutional Review Board (IRB) approval (IRB protocol number 201102270). MM cell lines were cultured in RPMI-1640 media (Corning, Tewksbury, MA) supplemented with 10% fetal bovine serum (FBS; Gibco, Life Technologies, Grand Island, NY), 2 mmol/L L-glutamine, 100 μg/mL penicillin, and 100 μg/mL streptomycin (Corning CellGro). HUVECs were cultured in Endothelial Growth Medium (EGM, Angio-Proteomie, Boston, MA) supplemented with endothelial growth supplements (including 10% FBS, recombinant growth factors, and 1% penicillin and streptomycin). All cells were cultured at 37 °C and in 5% CO_2_ in a NuAire water jacket incubator (Plymouth, MN).

### Expression of P-selectin in MM-associated ECs in human samples

BMMNCs from healthy (*n* = 6) and MM patients (*n* = 6) were washed with PBS and stained with anti-human CD45 (REA747), CD31 (REA730), VEGFR-2 (REA1046), and CD62P (REA389) mAbs or their respective isotype controls for 1 h. Cells were washed and analyzed by flow cytometry. First, we gated CD45-negative cells to exclude the leukocytes. Then from that population, we gated the ECs as CD31+/VEGFR-2+ double-positive cells. ECs were analyzed then for the expression of P-selectin (CD62P) as the ratio of the mean fluorescence intensity (MFI) of CD62P divided by the isotype control (RMFI). Then the RMFI values of P-selectin expression on ECs in MM patients were divided by the values acquired from the healthy donor samples^27^. Flow cytometry files were analyzed using MACSQuantify and FlowJo.

### Expression of P-selectin in ECs in vivo

All animal work presented in this study was approved by the Institutional Animal Care and Use Committee at Washington University School of Medicine in St. Louis. The mice used were NCG male 50–56-day-old mice (Charles River, Wilmington, MA) unless otherwise stated. MM.1S cells (2 × 10^6^ cells/mouse) were injected intravenously into five mice and tumor progression was confirmed using bioluminescent imaging (BLI) at 4 weeks post-injection. Five healthy mice were used as well to assess P-selectin expression. Mice were sacrificed and femurs were crushed and washed with PBS for the collection of BMMNCs. The cells were stained with CD45 (REA737), CD31 (390), VEGFR-2 (REA1116), and CD62P (REA344) murine mAbs or their respective isotype controls for 1 h. Cells were washed and analyzed by flow cytometry. ECs were gated as CD31 and VEGFR-2 positive cells. Then the RMFI values of P-selectin expression on ECs in MM-bearing mice were divided by the values acquired from the healthy mice.

For immunofluorescence, MM.1 S cells (2 × 10^6^ cells/mouse) were injected intravenously into three mice and tumor progression was confirmed using BLI at 4 weeks post-injection. Three healthy mice were used as well to assess VEGFR-2 and P-selectin expression. Mice were sacrificed; femurs were processed and embedded in paraffin^28^. Briefly, the processed slides were heated at 55 °C overnight to remove the paraffin and incubated in xylene twice for 5 min. To rehydrate the tissue, the slides were put into 100% ethanol twice for 3 min. Then 95% ethanol twice for 3 min and 80% ethanol twice for 3 min. The slides were then washed in PBS for 5 min and soaked in double-distilled water for 30 min. Antigen retrieval process was started by inserting 800 μL of unmasking solution (Vector Labs, Burlingame, CA) into 80 mL of double-distilled water. The slides were put into this solution, put into a pressure cooker, and microwaved on high for 8 min and medium for 3 min. The slides were set to cool in an unmasking solution for 20 min and washed in PBS for 5 min. The slides were then blocked with 120 mL of PBS supplemented with 0.3% Triton-x, 5% goat serum, and 5% FBS for 2 h at room temperature. Following blocking, we washed with PBS for 5 min and incubated the slides in the primary antibodies (VEGFR-2 (REA1116), and CD62P (RB40.34; BD Biosciences, San Jose, CA)) diluted in PBS supplemented with 3% bovine serum albumin. Each antibody concentration was ten micrograms per milliliter. The slides were incubated with the antibodies overnight in 4 °C. PBS was used to wash the slides three times for 5 min. A coverslip was then added with mounting medium for imaging. For imaging, we used an Olympus BX51 fluorescent microscope at Ex/em 365/420 and 620/670 nm.

### Tube-like formation of ECs on 2D Matrigel and 3DTEBM

On the first day, DiO-labeled MM cells (3 × 10^4^ cells/well) were cultured in regular 96-well plates (2D) or in the 3DTEBM^15^. To make the 3DTEBM, 40 µL of BM plasma was added to 60 µL RPMI-1640 complete medium with a final concentration of 1 and 4 mg/mL calcium chloride and tranexamic acid, respectively. On the next day, Matrigel was added on top of the 3DTEBM, or to the regular 96-well, as a control (2D). HUVECs were stained with DiD and plated on top of the Matrigel for all well conditions (2D and 3DTEBM; 3 × 10^4^ cells/well), in RPMI-1640 media without serum. The samples were imaged via ZEN 2009 using a fluorescent microscope 4 h post-addition of the HUVECs. Tube length was measured using ImageJ.

### Expression of P-selectin in ECs cultured with cell lines in vitro

The expression of P-selectin in vitro was evaluated in HUVECs using two models. (i) In the 2D tissue culture model, HUVECs (1 × 10^4^ cells pre-labeled with DiO) and MSP-1 stromal cells (1 × 10^4^ cells) were co-cultured with or without MM cell lines (MM.1S, H929, or OPM-2; 3 × 10^4^ cells) per well in a 96-well plate. (ii) In the 3DTEBM model, MSP-1 stromal cells (1 × 10^4^ cells) with or without MM cell lines (MM.1S, H929, or OPM-2; 3 × 10^4^ cells) were suspended in BM plasma and set to solidify into a 3DTEBM matrix. After 2 h, Matrigel (Corning, Tewksbury, MA) was added on top of the scaffold, and HUVECs (1 × 10^4^ cells pre-labeled with DiO) were added on top of the Matrigel with non-supplemented EGM media. HUVECs and stromal cells with and without MM cells were cultured in 2D and 3DTEBM models for 24 h. Then, the cultures were digested with collagenase, and cells were retrieved for flow cytometry analysis. The experiment was independently repeated three times with five replicates each.

To determine that the increase in P-selectin expression on ECs was specifically associated with the presence of MM, we co-cultured ECs with MM cells or normal peripheral blood mononuclear cells (PBMCs; 3 × 10^4^ cells/well) extracted from healthy donors, in the 3DTEBM following the procedure described above. For the effect of Y27632 on the expression of P-selectin, HUVECs were plated on the 3DTEBM containing MM and treated with free Y27632 (25 μM) for 24 and 48 h and analyzed for P-selectin expression via flow cytometry.

### Confocal imaging of 3DTEBM cultures of HUVECs

MSP-1 stromal cells (1 × 10^4^ cells pre-labeled with DiD) and MM.1S cells (3 × 10^4^ cells pre-labeled with DiO) were suspended in BM plasma to form a 3D scaffold in a Nunc Lab-Tek II Chamber Slide System (Thermo Fisher, Waltham, MA). After 2 h, Matrigel was added on top of the scaffold and HUVECs (1 × 10^4^ cells pre-labeled with calcein violet) were subsequently added on top of the Matrigel. The HUVECs, stromal cells, and MM cells were cultured in the 3DTEBM for 24 h. Samples were then imaged using a FV1000 confocal microscope with an XLUMPLFLN 20XW/1.0 immersion objective lens (Olympus, Central Valley, PA) (excitation/emission: 405/450 ± 20 (calcein violet), 488/ 520 ± 20 (DiO), and 633/650 + (DiD) nm).

### Preparation and characterization of liposomes

Liposomes were prepared using the thin layer evaporation method^29^. Briefly, lipids (DPPC, Chol, DSPE-mPEG2000, and DSPE-PEG(2000)-succinyl at a molar ratio of 6:3:0.5:0.5) were dissolved in a chloroform/methanol mixture (3:1, v/v) and the solvent was then evaporated through a rotary evaporator (Heidolph, Schwabach, Germany) to form a thin lipid film. The film was then hydrated with PBS and extruded with an extruder set (Avanti Polar Lipids). Fluorescent liposomes were prepared by dissolving DiD in the organic solvent with the lipids (before film formation).

Conjugation of PSGL-1 to the surface of liposomes was performed by using carbodiimide chemistry. Briefly, the liposomes were suspended in a solution of 0.25 M EDC and 0.25 M NHS (in water) and incubated for 10 min at room temperature. Then, PSGL-1 was added to the mixture and the colloidal suspension was incubated at 4 °C overnight in a light-protected environment with gentle-stirring. Unbound protein was removed using Amicon Ultra Centrifugal Filter Units (100 kDa MWCO). The mean sizes, polydispersity index (PDI) and zeta-potential (ZP) were analyzed by dynamic light scattering (DLS) analysis using a Zetasizer Nano ZS (Malvern, Malvern, UK).

### Cryogenic transmission electron microscopy imaging of liposomes

Samples were prepared on Quantifoil holey carbon grids (R2/2 300 mesh copper) and plunge frozen using a Vitrobot Mark IV (Thermo Fisher) which was set to 4 °C and 100% humidity. Three microliters of sample was applied to the Quantifoil grids, blotted to remove excess fluid, and plunge frozen into liquid ethane. Grids were then stored in liquid nitrogen until imaged. Vitrified grids were imaged using a JEM-1400 TEM (JEOL, Peabody, MA) operating at 120 kV, equipped with a 626 single tilt liquid nitrogen cryo-transfer holder (Gatan, Pleasanton, CA) and a XR111 CCD camera (Advanced Microscopy Techniques, Woburn, MA). The sample holder was kept at −175 °C during imaging to prevent devitrification. All images were acquired using a nominal magnification of ×25,000 corresponding to a pixel size of 7.38 Å/pixel.

### Measurement of the affinity of PSGL-1-targeted liposomes for P-selectin by surface plasmon resonance

The affinity of PSGL-1-targeted liposomes for P-selectin was measured by the biosensor-based surface plasmon resonance (SPR) technique using an automatic apparatus BIAcore T200 (GE Healthcare, Chicago, IL). Recombinant P-selectin protein was immobilized using carbodiimide chemistry on the CM4 sensor chip surface (ligand), and PSGL-1-targeted and non-targeted liposomes were used as the analyte.

### Binding of PSGL-1-targeted liposomes to ECs in vitro

HUVECs (pre-labeled with DiO) were grown on top of the 3DTEBM as described above. DiD-labeled non-targeted or PSGL-1-targeted liposomes were cultured with the HUVECs for 2 h. The 3D cultures were then digested, washed, and analyzed via flow cytometry. The experiment was independently repeated three times with five replicates each.

### Binding of PSGL-1-targeted liposomes to ECs in vivo

MM.1 S cells (2 × 10^6^ cells/mouse) were injected intravenously into ten mice and tumor progression was confirmed using BLI at 4 weeks post-injection. Mice were then injected intravenously with DiD-labeled non-targeted or PSGL-1-targeted liposomes (2 mg/mL of lipids; 5 mice per group). Mice were sacrificed and femurs were flushed with PBS and crushed. The cells were stained with CD45 (REA737), CD31 (390), and VEGFR-2 (REA1116) murine mAbs or their respective isotype controls for 1 h. Cells were washed and analyzed by flow cytometry. ECs were gated as CD31 and VEGFR-2 positive cells. Then the MFI values of the PSGL-1-targeted liposomes bound to ECs in mice were divided by the values obtained from the non-targeted samples.

### Fluorescent imaging of tumor in vivo and organs ex vivo

Four NSG mice (shaven) were injected intravenously with non-targeted and PSGL-1-targeted liposomes that were stained with DiO and DiD, respectively. These mice were imaged at *t* = 0.1, 1, 4, and 24 h using the In-Vivo MS FX PRO fluorescent imaging system (Bruker, Billerica, MA) at Ex/em of 460/535 and 620/670 nm. Following 24 h, the tissues were extracted from each mouse and imaged in the In-Vivo MS FX PRO fluorescent Bruker imaging system.

### Histological assessments of tissue

Following ex vivo imaging, tissues were frozen in OCT (Tissue Tek, Torrance, CA), sliced using a Cryocut 1800 (Leica, Wetzlar, Germany), and imaged using an Olympus BX51 fluorescent microscope at Ex/em 460/535 and 620/670 nm. No staining was used due to the fluorescence of the liposomes.

### High-performance liquid chromatography for detection of BTZ and Y27632

BTZ and Y27632 were analyzed using high-performance liquid chromatography (HPLC, Agilent 1100 series, Santa Clara, CA) with a reverse phase C-18 column (Agilent Zorbax Eclipse XDB-C18, 4.6 mm × 150 mm).

For detection of BTZ, a 50% acetonitrile solution in water containing 0.1% trifluoroacetic acid (TFA) was used as the mobile phase at a flow rate of 1 mL/min, as reported previously^14^. A calibration curve was obtained by plotting the area under the curve (AUC) of the BTZ HPLC peak (at retention time = 2.2 min, *λ* = 270 nm) for a concentration range of 0–200 μg/mL.

For detection of Y27632, a gradient of acetonitrile/water containing 0.1% TFA was used as the mobile phase at a flow rate of 1 mL/min^30^. The percent of acetonitrile in the mobile phase was 0% (at 0–3 min), then decreased gradually to 33% water (3–3.5 min), and decreased gradually back to 0% (3.5–7 min). A calibration curve was obtained by plotting the AUC of the Y27632 HPLC peak (at retention time = 4 min, *λ* = 260 nm) for a concentration range of 0–200 μg/mL. All HPLC data were analyzed via OpenLab.

### Drug release and evaluation of drug entrapment efficiency

Each drug was encapsulated in a separate liposome formulation followed by centrifugation (130,000 rcf at 4 °C for 1 h) to remove any free drug and re-suspended in fresh PBS. BTZ liposomes were put into dialysis bags (3.5 kD; Spectrum Labs, Rancho Dominguez, CA); the supernatant was taken for each time point (*t* = 1, 3, 6, 12, 24, 48) and analyzed using the HPLC methods mentioned above.

To evaluate loading efficiency, liposomes were centrifuged at 130,000 rcf at 4 °C for 1 h using a Beckman Optima™ XPN ultracentrifuge equipped with a SW 50.1 fixed angle rotor (Beckman Coulter Inc., Fullerton, CA, USA). The amount of BTZ and Y27632 in the supernatant was evaluated by HPLC. The entrapment efficiency (EE) was calculated according to the following equation:

1 $${\mathrm{EE}} = D_{\mathrm{T}} - D_{\mathrm{U}}/D_{\mathrm{T}} \times 100$$

where *D*_T_ is the total amount of drug added to the formulation during the preparation, and *D*_U_ is the amount of unincorporated drug found in the supernatant.

### Effect of free and liposomal Y27632 on trans-endothelial migration of MM cells in vitro

Trans-endothelial migration^5^ was performed by incubating HUVECs (5 × 10^3^ cells) overnight in the upper chamber of a Boyden chamber (Corning) prior to adhesion assay. MM.1S cells were pre-treated with (or without) free Y27632 (25 μM) or liposomal Y27632 (25 μM equivalent) for 3 h. Cells were then placed in the upper migration chamber in the presence or absence of 30 nM SDF-1 in the lower chamber. After 3 h of incubation, cells that migrated to the lower chambers were counted by flow cytometry.

### Effect of free and liposomal Y27632 on mobilization of MM cells to the circulation in vivo

MM.1S-GFP-Luc cells (2 × 10^6^ cells/mouse) were injected intravenously into nine mice, and tumor progression was confirmed using BLI at 4 weeks post-injection. Mice were treated with intravenous injections of (i) free Y27632 (2.5 mg/kg, *n* = 3); (ii) Y27632-loaded non-targeted liposomes (2.5 mg/kg equivalent, *n* = 3); and (iii) Y27632-loaded PSGL-1-targeted liposomes (2.5 mg/kg, *n* = 3). Blood was collected from the tail vein of each mouse at 0 (before), 2, 4, and 24 h after injection. Blood samples were lysed with 1X red blood cell lysis buffer (BioLegend, San Diego, CA) using the manufacturer’s instructions and PBMCs were analyzed via flow cytometry.

### Effect of free and liposomal drugs on cell signaling in MM cells and ECs

HUVECs were treated with vehicle (control), free Y27632 (25 μM), empty liposomes, and liposomal Y27632 (25 μM) overnight and then cultured with MM.1S for 6 h in serum-free media. The MM cells were then separated from the HUVECs by gently tapping the culture plate multiple times and washing the HUVECs with PBS to separate the floating MM cells from the adherent HUVECs. Proteins were then extracted from each cell type and subjected to immunoblotting for pSRC, pFAK, and α-Tubulin (60, 125, and 52 kDa, respectively).

MM.1 S cells were cultured and treated with vehicle (control), free BTZ (5 nM), empty liposomes, and liposomal BTZ (5 nM) for 24 h. Proteins were then extracted and subjected to immunoblotting for p21, pRB, cPARP, cCasp3, cCasp9, pAKT, pS6R, pERK, and α-Tubulin (21, 110, 89, 17, 37, 60, 32, 140, and 52 kDa, respectively).

For immunoblotting, we cut the membranes according to the target protein size before antibody staining. Cells were lysed with 1X lysis buffer (Cell Signaling, #9803). The protein concentration was determined by Bradford assay (Bio-Rad, Hercules, CA), and 50 μg of protein was loaded per lane. Proteins were separated by electrophoresis using NuPAGE 4–12% Bis-Tris gels (Novex, Life Technologies, Grand Island, NY) and transferred to a nitrocellulose membrane using an iBlot system (Invitrogen). Membranes were blocked with 5% non-fat milk in Tris-buffered saline/Tween-20 (TBST) buffer and incubated with primary antibodies overnight at 4 °C. The membranes were then washed with TBST for 30 min, incubated for 1 h at room temperature with horseradish peroxidase (HRP)-conjugated secondary antibody, washed, and developed using a Novex ECL Plus Chemiluminescent Kit (Thermo Fisher). Blots were imaged on a ChemiDoc XRS imaging system via Bio-Rad Image Lab (Bio-Rad).

### Effect of free and liposomal BTZ on MM and EC viability in vitro

DiD-labeled HUVECs and DiO-labeled MM.1S cells were co-cultured overnight and treated with vehicle (control), free BTZ (0–50 nM), empty liposomes, or liposomal Y27632 (0–50 nM equivalent) for 24 h, and the survival of MM and HUVECs was analyzed via flow cytometry. MM cells were gated as DiO+ cells and HUVECs were gated as DiD+ cells, and each cell population was counted and normalized against counting beads (Invitrogen). Survival was calculated as the percent of vehicle-treated controls.

### Efficacy of BTZ and Y27632-loaded PSGL-1-targeted liposomes on MM tumor progression in vivo

MM.1S-GFP-Luc cells (2 × 10^6^ cells/mouse) were injected intravenously into 84 mice, and tumor progression was confirmed using BLI at 3 weeks post-injection (average photon flux of 2.5E07 photons per second). Mice were randomized into 12 groups of 7 mice each, which received weekly intravenous injections of (i) saline, (ii) Y27632 as a free drug (2.5 mg/kg), (iii) BTZ as a free drug (1 mg/kg), (iv) a combination of free BTZ and free Y27632, (v) empty non-targeted liposomes, (vi) non-targeted liposomal Y27632 (2.5 mg/kg equivalent), (vii) non-targeted liposomal BTZ (1 mg/kg), (viii) non-targeted liposomal combination of BTZ and Y27632 in the same liposome (2.5 mg/kg and 1 mg/kg, respectively), (ix) empty PSGL-1-targeted liposomes, (x) PSGL-1-targeted liposomal Y27632 (2.5 mg/kg), (xi) PSGL-1-targeted liposomal BTZ (1 mg/kg), and (xii) PSGL-1-targeted liposomal combination of BTZ and Y27632 in the same liposome (2.5 mg/kg and 1 mg/kg, respectively). Tumor progression was assessed weekly by BLI. Weight was recorded twice per week, and the survival and general health of the mice were recorded daily.

### Statistical analyses

All data from in vitro and in vivo experiments were expressed as means ± standard deviation. Statistical significance was analyzed using a Student’s *t*-test or two-way analysis of variance (ANOVA). Log-rank test was used to compare the Kaplan–Meier curves. *P* values <0.05 were used to indicate statistically significant differences. All graphs were plotted on Microsoft Excel and GraphPad Prism.

### Reporting summary

Further information on research design is available in the Nature Research Reporting Summary linked to this article.

## Supplementary information

Supplementary Information

Reporting Summary

## Data Availability

Source data are included in the supplementary. All data files generated are available upon request. Source data are provided with this paper.

## Source data

Source Data
