# Supplementary material for: Tumor microenvironment-targeted nanoparticles loaded with bortezomib and ROCK inhibitor improve efficacy in multiple myeloma
Source: Nat Commun. 2020 Nov 27;11:6037. doi: 10.1038/s41467-020-19932-1 (PMC7699624; doi:10.1038/s41467-020-19932-1)
Supplement: Supplementary file 1 — Supplementary Information [file 41467_2020_19932_MOESM1_ESM.pdf]

Supplementary Figure 1

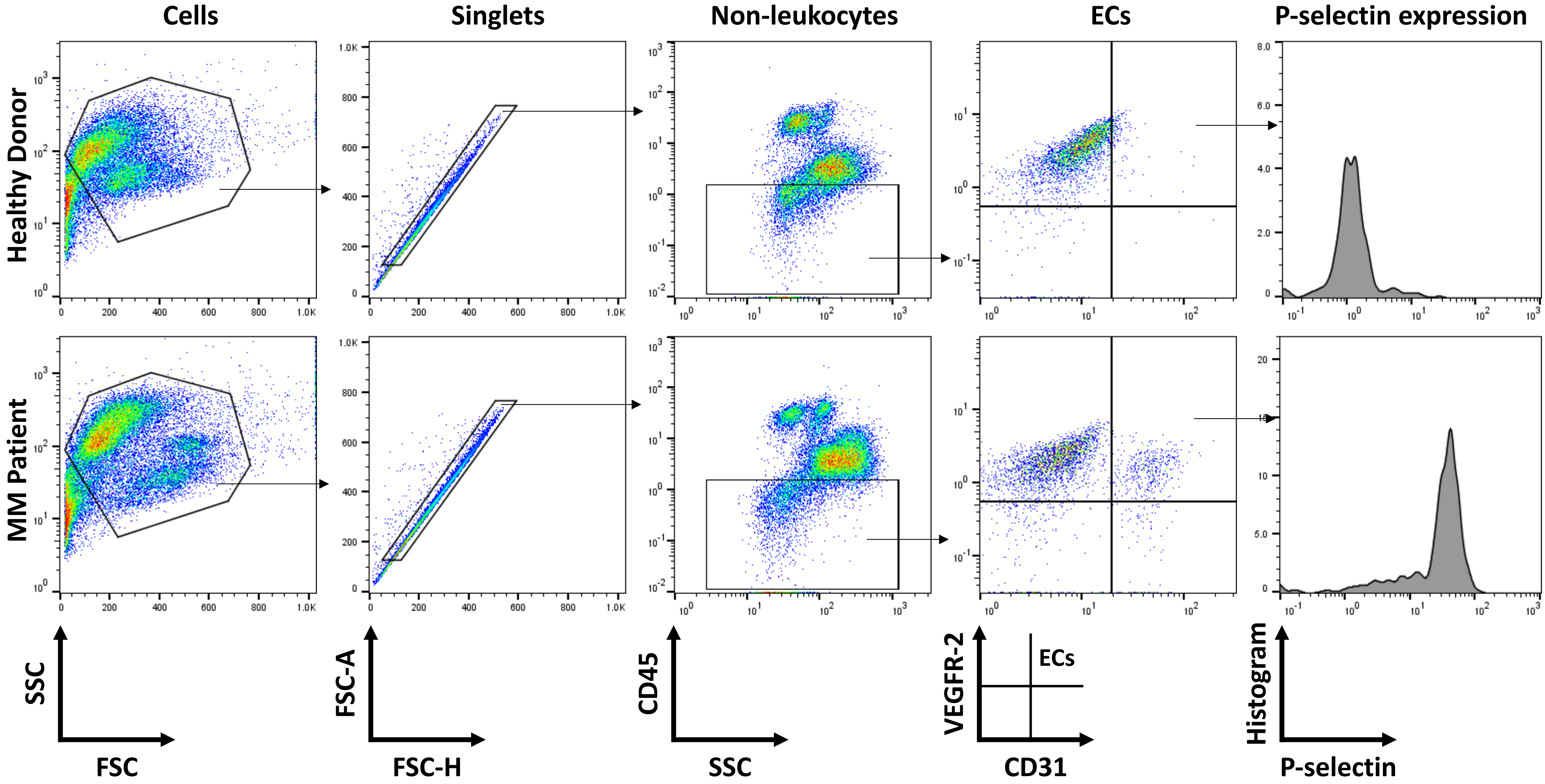

Supplementary Fig. 1. Gating strategy used for Figures 1a and b.

Supplementary Figure 2

a

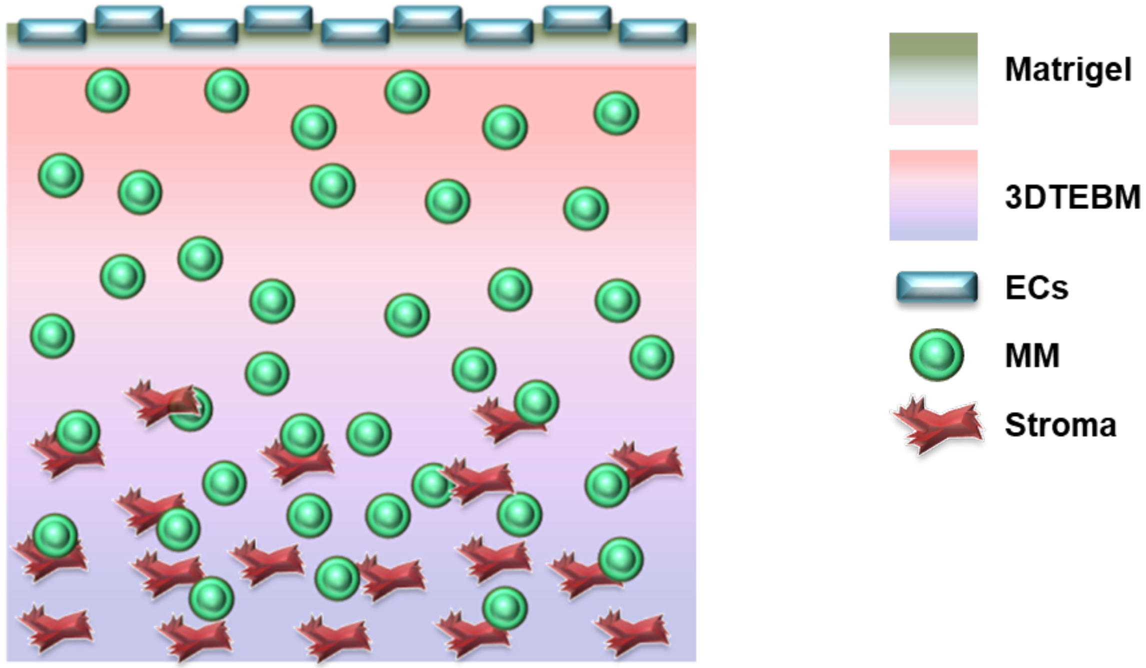

b

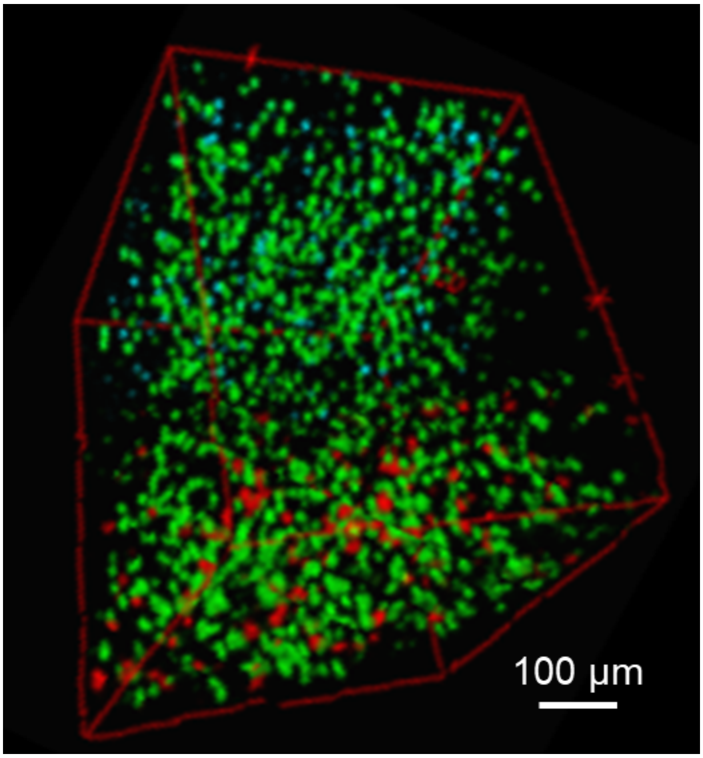

Supplementary Fig. 2. Illustrations of patient-derived 3D tissue-engineered bone marrow (3DTEBM). a MM and stromal cells were cultured inside 3DTEBM. Endothelial cells (ECs) were incubated on top of the 3DTEBM with Matrigel. **b** Confocal microscopy image of MM (green) and MM-derived stroma (red) inside our patient-derived 3DTEBM with ECs (cyan) cultured on top.

Supplementary Figure 3

a

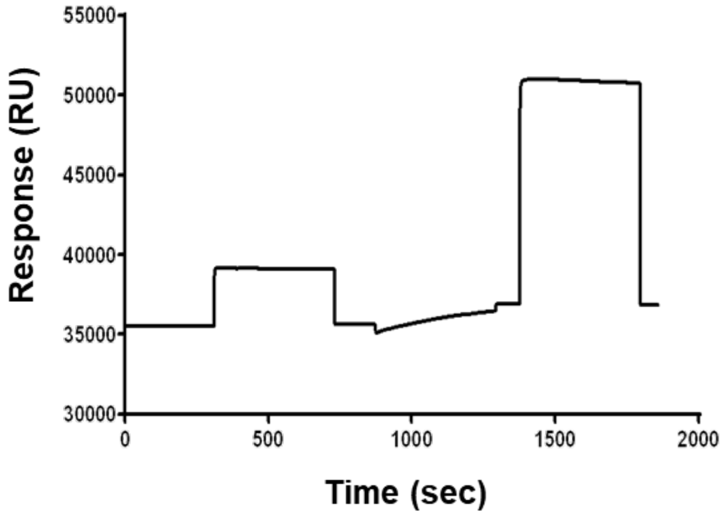

Supplementary Fig. 3. Immobilization of P-selectin. Recombinant P-selectin was immobilized onto the surface plasmon resonance sensor chip via amine coupling.

Supplementary Figure 4

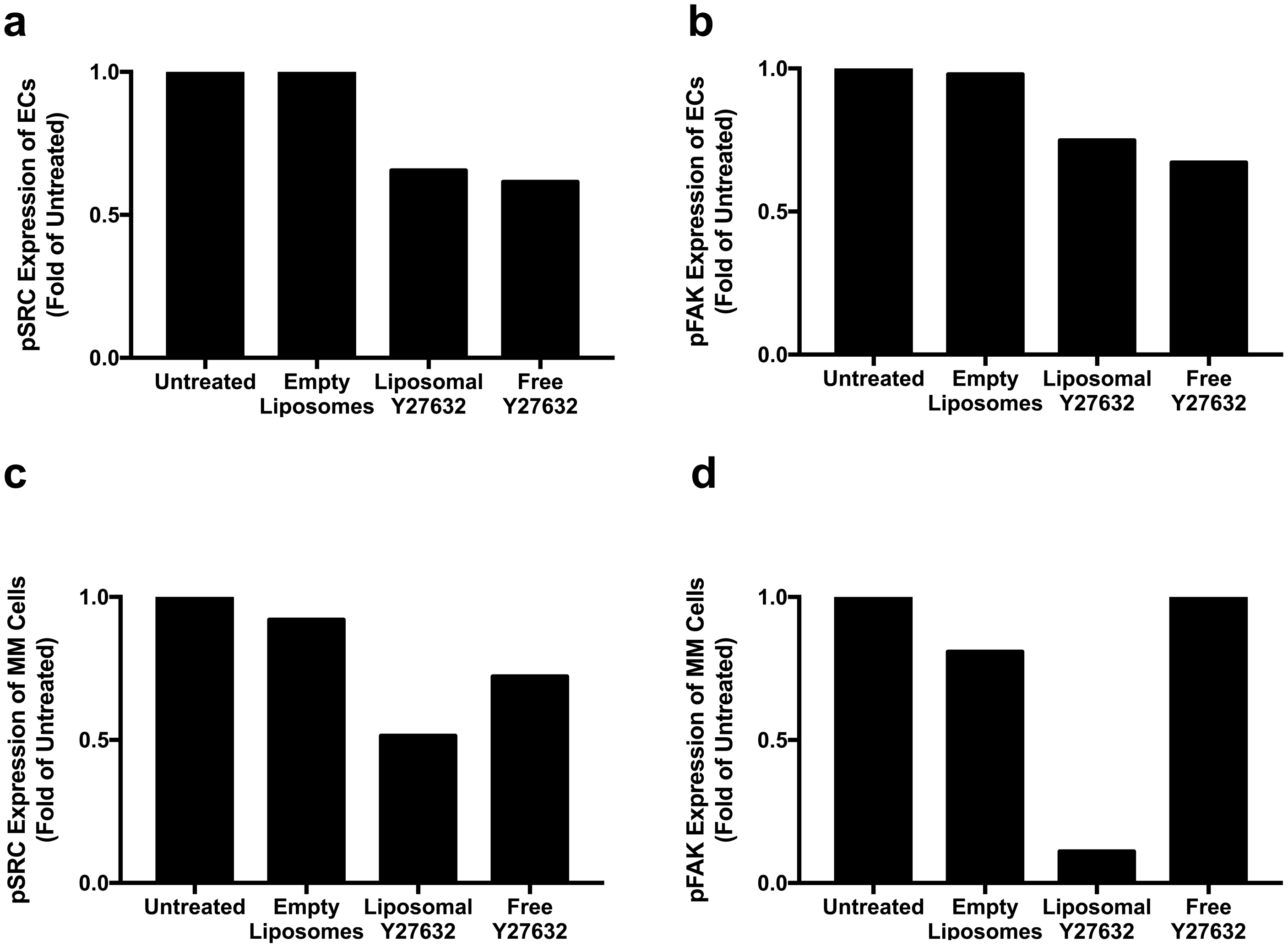

Supplementary Fig. 4. Quantitative analysis of cytoskeletal signaling. The effect of liposomal and free Y27632 on **a** pSRC and **b** pFAK in ECs. The effect of liposomal and free Y27632 on **c** pSRC and **d** pFAK in MM cells.

Supplementary Figure 5

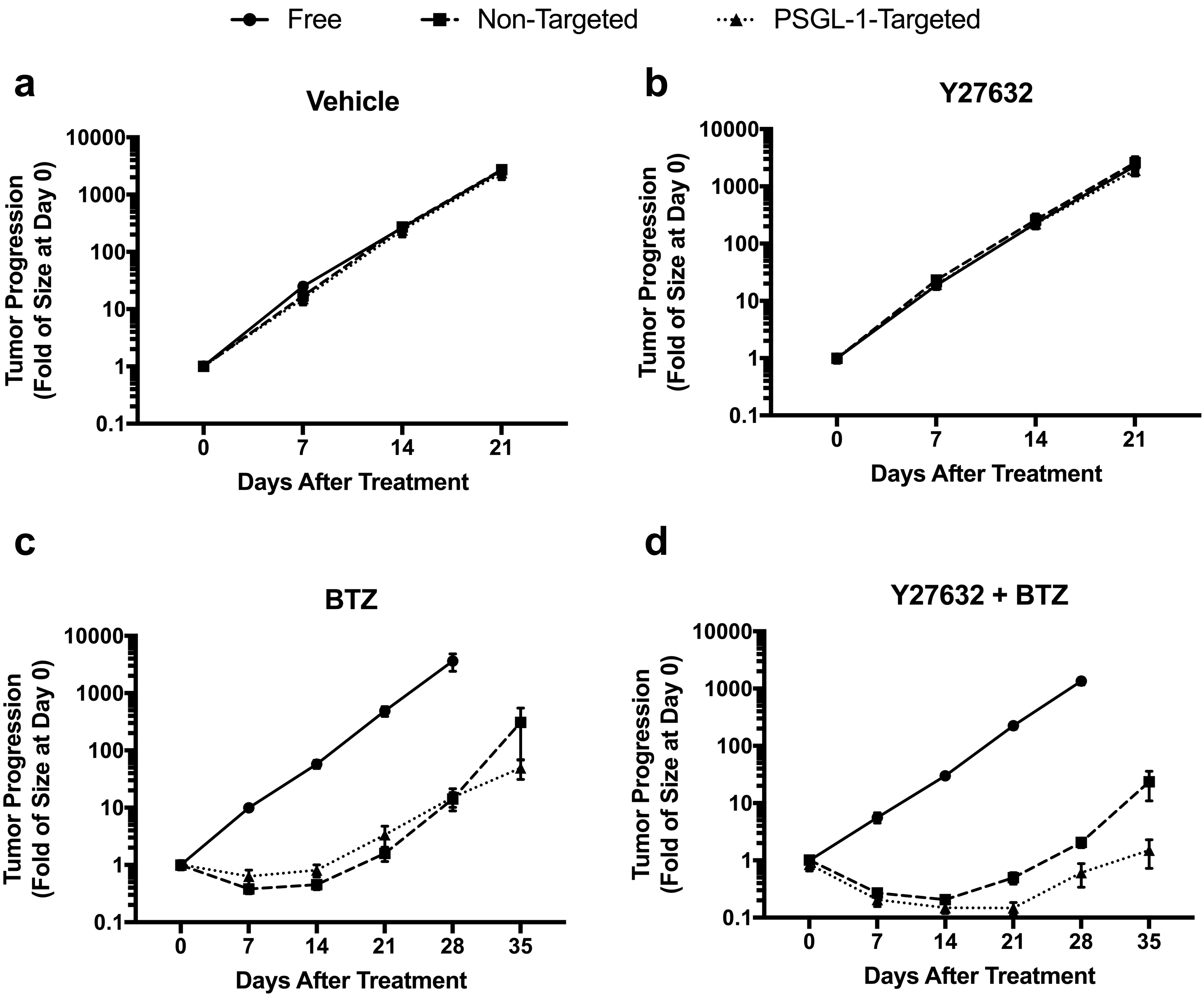

Supplementary Fig. 5. Tumor progression stratified by treatment rather than vehicle delivery in vivo. Tumor burden after treatment with free drug, non-targeted, and PSGL-1-targeted liposome formulations for **a** vehicle, **b** Y27632, **c** BTZ, and **d** combination treatments.

Supplementary Figure 6

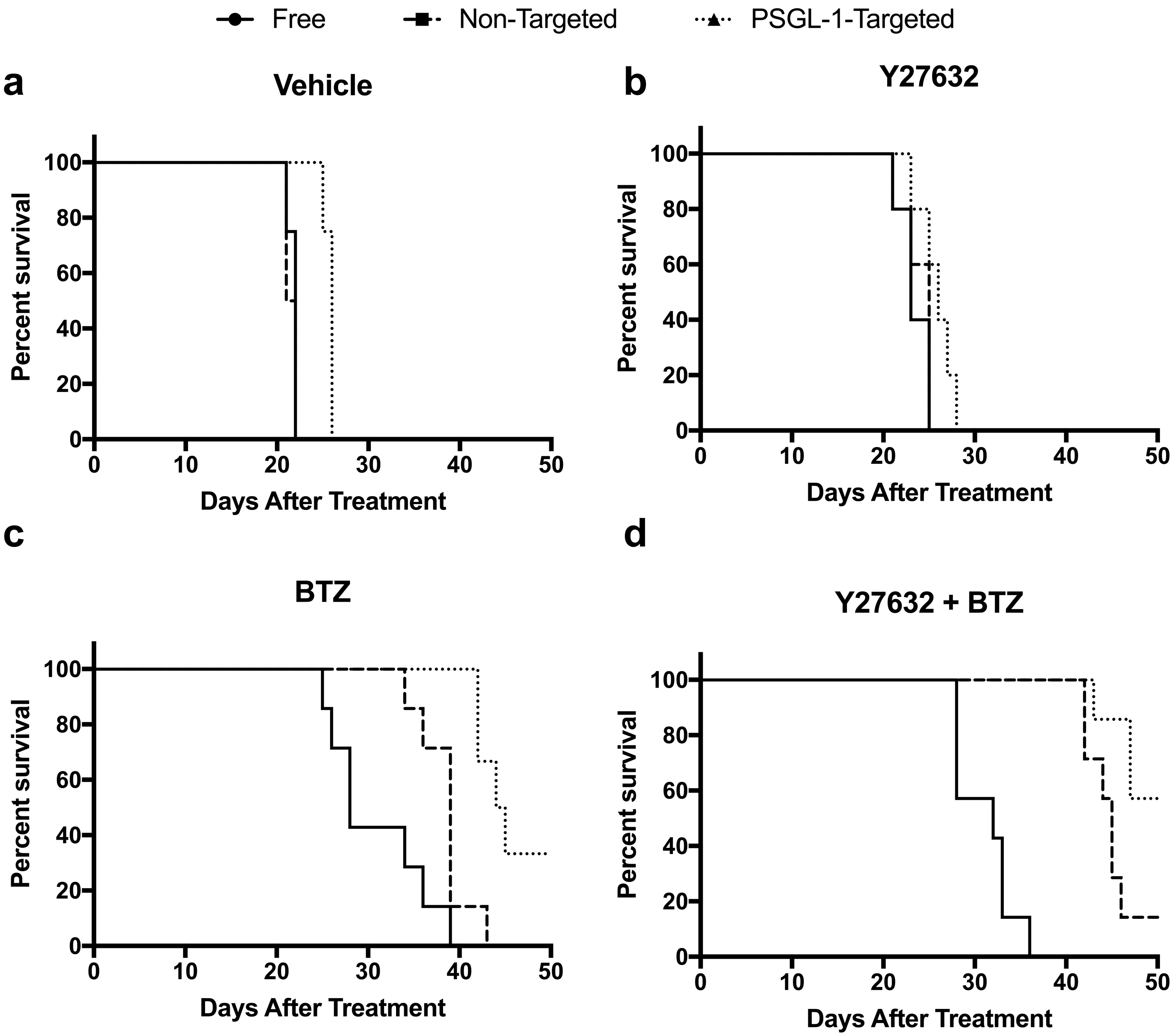

Supplementary Fig. 6. Survival stratified by treatment rather than vehicle delivery in vivo. Percent survival after treatment with free drug, non-targeted, and PSGL-1-targeted liposome formulations for **a** vehicle, **b** Y27632, **c** BTZ, and **d** combination treatments.

Supplementary Table 1. Parameters for liposomes used in vitro.

Formulation	Mean Size (nm)	Polydispersity Index	Zeta Potential (mV)
Non-Targeted	148.4 ± 1.248	0.070 ± 0.015	- 41.9 ± 0.192
PSGL-1-Targeted	146.9 ± 0.885	0.081 ± 0.021	- 36.1 ± 0.781

Supplementary Table 2. Parameters for liposomes used in vivo.

Formulation	Mean Size (nm)	Polydispersity Index	Zeta Potential (mV)
Non-Targeted Vehicle	146.3 ± 1.914	0.153 ± 0.010	- 44.4 ± 0.50
Non-Targeted Y27632	138.3 ± 1.027	0.051 ± 0.009	- 32.1 ± 0.06
Non-Targeted BTZ	168.2 ± 1.007	0.109 ± 0.006	- 45.9 ± 1.29
Non-Targeted Y27632 + BTZ	163.2 ± 2.372	0.126 ± 0.017	- 41.4 ± 1.42
PSGL-1-Targeted Vehicle	147.7 ± 0.9960	0.157 ± 0.017	- 33.9 ± 1.27
PSGL-1-Targeted Y27632	172.0 ± 2.826	0.101 ± 0.037	- 35.4 ± 2.86
PSGL-1-Targeted BTZ	154.0 ± 3.947	0.095 ± 0.021	- 42.0 ± 1.91
PSGL-1-Targeted Y27632 + BTZ	172.6 ± 4.883	0.129 ± 0.027	- 29.4 ± 0.88

Mean ± standard deviation.

	Treatment	P-value (Efficacy)	P-value (Survival)
Free	Vehicle vs. Y27632	.556	.102
	Vehicle vs. BTZ	.005	.005
	Vehicle vs. Y27632 + BTZ	<.001	.005
	Y27632 vs. BTZ	.005	.005
	Y27632 vs. Y27632 + BTZ	<.001	.002
	BTZ vs. Y27632 + BTZ	.120	.855
Non-Targeted	Vehicle vs. Y27632	.851	.069
	Vehicle vs. BTZ	<.001	<.001
	Vehicle vs. Y27632 + BTZ	<.001	.003
	Y27632 vs. BTZ	<.001	.003
	Y27632 vs. Y27632 + BTZ	<.001	.003
	BTZ vs. Y27632 + BTZ	<.001	.005
PSGL-1-Targeted	Vehicle vs. Y27632	.445	.974
	Vehicle vs. BTZ	<.001	.009
	Vehicle vs. Y27632 + BTZ	<.001	.008
	Y27632 vs. BTZ	<.001	.002
	Y27632 vs. Y27632 + BTZ	<.001	.001
	BTZ vs. Y27632 + BTZ	<.001	.276

Supplementary Table 3. Statistical analyses of in vivo dataset in Fig. 7. Data from tumor efficacy experiment were analyzed using two-way analysis of variance with Tukey's multiple comparisons test. Data from survival were analyzed using Kaplan-Meier and logrank test.

	Treatment	P-value (Efficacy)	P-value (Survival)
Vehicle	Free vs. Non-Targeted	.606	.495
	Free vs. PSGL-1-Targeted	.362	.451
	Non-Targeted vs. PSGL-1-Targeted	.989	.458
Y27632	Free vs. Non-Targeted	.943	.614
	Free vs. PSGL-1-Targeted	.865	.658
	Non-Targeted vs. PSGL-1-Targeted	.940	.604
BTZ	Free vs. Non-Targeted	<.001	.003
	Free vs. PSGL-1-Targeted	<.001	<.001
	Non-Targeted vs. PSGL-1-Targeted	.897	.003
Y27632 + BTZ	Free vs. Non-Targeted	<.001	<.001
	Free vs. PSGL-1-Targeted	<.001	<.001
	Non-Targeted vs. PSGL-1-Targeted	.025	.016

Supplementary Table 4. Statistical analyses of in vivo dataset in Supplementary Figs. 5 and 6. Data from tumor efficacy experiment were analyzed using two-way analysis of variance with Tukey's multiple comparisons test. Data from survival were analyzed using Kaplan-Meier and logrank test.
