# Supplementary material for: Tumor microenvironment-targeted nanoparticles loaded with bortezomib and ROCK inhibitor improve efficacy in multiple myeloma
Source: Nat Commun. 2020 Nov 27;11:6037. doi: 10.1038/s41467-020-19932-1 (PMC7699624; doi:10.1038/s41467-020-19932-1)
Supplement: Supplementary file 2 — Reporting Summary [file 41467_2020_19932_MOESM2_ESM.pdf]

nature research

Corresponding author(s): Abdel Kareem Azab

Last updated by author(s): Sep 28, 2020

Reporting Summary

Nature Research wishes to improve the reproducibility of the work that we publish. This form provides structure for consistency and transparency in reporting. For further information on Nature Research policies, see Authors & Referees and the Editorial Policy Checklist .

Statistics

For all statistical analyses, confirm that the following items are present in the figure legend, table legend, main text, or Methods section.

n/a	Confirmed	
☐	☒	The exact sample size (*n*) for each experimental group/condition, given as a discrete number and unit of measurement
☐	☒	A statement on whether measurements were taken from distinct samples or whether the same sample was measured repeatedly
☐	☒	The statistical test(s) used AND whether they are one- or two-sided<br>*Only common tests should be described solely by name; describe more complex techniques in the Methods section.*
☒	☐	A description of all covariates tested
☒	☐	A description of any assumptions or corrections, such as tests of normality and adjustment for multiple comparisons
☐	☒	A full description of the statistical parameters including central tendency (e.g. means) or other basic estimates (e.g. regression coefficient) AND variation (e.g. standard deviation) or associated estimates of uncertainty (e.g. confidence intervals)
☐	☒	For null hypothesis testing, the test statistic (e.g. *F*, *t*, *r*) with confidence intervals, effect sizes, degrees of freedom and *P* value noted<br>*Give P values as exact values whenever suitable.*
☒	☐	For Bayesian analysis, information on the choice of priors and Markov chain Monte Carlo settings
☒	☐	For hierarchical and complex designs, identification of the appropriate level for tests and full reporting of outcomes
☒	☐	Estimates of effect sizes (e.g. Cohen's *d*, Pearson's *r*), indicating how they were calculated

Our web collection on statistics for biologists contains articles on many of the points above.

Software and code

Policy information about availability of computer code

Data collection	MACSQuantify V2.11, ZEN 2009 V5.5, BIAcore T200 V2.0, OpenLab V2.5, ImageJ V1.53, and Bio-Rad Image Lab V6.1 softwares
Data analysis	Microsoft Excel 2016, FlowJo V10.7, GraphPad Prism V8.4.3.

For manuscripts utilizing custom algorithms or software that are central to the research but not yet described in published literature, software must be made available to editors/reviewers. We strongly encourage code deposition in a community repository (e.g. GitHub). See the Nature Research guidelines for submitting code & software for further information.

Data

Policy information about availability of data

All manuscripts must include a data availability statement. This statement should provide the following information, where applicable:

- Accession codes, unique identifiers, or web links for publicly available datasets
- A list of figures that have associated raw data
- A description of any restrictions on data availability

Source data is available in the supplementary material. Data produced and analyzed in this study are available upon request from the corresponding author.

Field-specific reporting

Please select the one below that is the best fit for your research. If you are not sure, read the appropriate sections before making your selection.

☒ Life sciences ☐ Behavioural & social sciences ☐ Ecological, evolutionary & environmental sciences

For a reference copy of the document with all sections, see nature.com/documents/nr-reporting-summary-flat.pdf

Life sciences study design

All studies must disclose on these points even when the disclosure is negative.

Sample size	Sample size was determined based on our experiences and literature. Relevant studies were cited where appropriate.
Data exclusions	Data were not excluded from the analyses performed in this study.
Replication	The number of repeats for each experiment was stated in the legend of each figure.
Randomization	All patient samples and mice were randomized prior to use.
Blinding	The investigators were not blinded to the treatment groups due to the responsibility of the investigators to treat the mice.

Reporting for specific materials, systems and methods

We require information from authors about some types of materials, experimental systems and methods used in many studies. Here, indicate whether each material, system or method listed is relevant to your study. If you are not sure if a list item applies to your research, read the appropriate section before selecting a response.

Materials & experimental systems

n/a	Involved in the study	
☐	☒	Antibodies
☐	☒	Eukaryotic cell lines
☒	☐	Palaeontology
☐	☒	Animals and other organisms
☐	☒	Human research participants
☒	☐	Clinical data

Methods

n/a	Involved in the study	
☒	☐	ChIP-seq
☐	☒	Flow cytometry
☒	☐	MRI-based neuroimaging

Antibodies

Antibodies used

Antibodies used for western blot were purchased from Cell Signaling Technology (Danvers, MA). Phospho-Akt (pAKT; #4060), phospho-Erk1/2 (pERK; #4370), phospho-Rb (pRB; #9308), p21 (#2947), cleaved Caspase3 (cCasp3; #9664), cleaved Caspase 9 (cCasp9; #7237), cleaved PARP (cPARP; #5625), phospho-FAK (pFAK; #3284), phospho-SRC (pSRC; #6943), and phospho-S6 ribosomal protein (pS6R; #4858) were all used at a dilution of 1:1000. α-Tubulin (#2125) and b-actin (#4970) were used as a loading controls at a dilution of 1:3000.

Antibodies used for flow cytometry were purchased from Miltenyi Biotec. Anti-human CD45 (REA747), CD31 (REA730), VEGFR-2 (REA1046) and CD62P (REA389). Anti-mouse CD45 (REA737), CD31 (390), VEGFR-2 (REA1116), and CD62P (REA344). These antibodies were not diluted and were used as recommended by the manufacturer.

Antibodies used for immunohistochemistry were purchased from Miltenyi Biotec (VEGFR-2; REA1116; 1/30) or BD Biosciences (CD62P; RB40.34; 1/30).

Validation

The above-mentioned antibodies were used based on instructions of manufacturers. Phospho-Akt (#4060, Cell Signaling, https://www.cellsignal.com/products/primary-antibodies/phospho-akt-ser473-d9e-xp-rabbit-mab/4060?Ntk=Products&Ntt=4060), phospho-Erk1/2 (#4370, Cell Signaling, https://www.cellsignal.com/products/primary-antibodies/phospho-p44-42-mapk-erk1-2-thr202-tyr204-d13-14-4e-xp-rabbit-mab/4370?Ntk=Products&Ntt=4370), phospho-Rb (#9308, Cell Signaling, https://www.cellsignal.com/products/primary-antibodies/phospho-rb-ser807-811-antibody/9308?Ntk=Products&Ntt=9308), p21 (#2947, Cell Signaling, https://www.cellsignal.com/products/primary-antibodies/p21-waf1-cip1-12d1-rabbit-mab/2947?Ntk=Products&Ntt=2947), cCasp3 (#9664, Cell Signaling, https://www.cellsignal.com/products/primary-antibodies/cleaved-caspase-3-asp175-5a1e-rabbit-mab/9664?Ntk=Products&Ntt=9664), cCasp9 (#7237, Cell Signaling, https://www.cellsignal.com/products/primary-antibodies/cleaved-caspase-9-asp330-d2d4-rabbit-mab/7237?Ntk=Products&Ntt=7237), cPARP (#5625, Cell Signaling, https://www.cellsignal.com/products/primary-antibodies/cleaved-parp-asp214-d64e10-xp-rabbit-mab/5625?Ntk=Products&Ntt=5625), pFAk (#3284, Cell Signaling, https://www.cellsignal.com/products/primary-antibodies/phospho-fak-tyr925-antibody/3284?Ntk=Products&Ntt=3284), pSRC (#6943, Cell Signaling, https://www.cellsignal.com/products/primary-antibodies/phospho-src-family-tyr416-d49g4-rabbit-mab/6943?Ntk=Products&Ntt=6943), pS6R (#4858, Cell Signaling, https://www.cellsignal.com/products/primary-antibodies/phospho-s6-ribosomal-protein-ser235-236-d57-2-2e-xp-rabbit-mab/4858?Ntk=Products&Ntt=4858), α-Tubulin (#2125, Cell Signaling, https://www.cellsignal.com/products/primary-antibodies/a-tubulin-11h10-rabbit-mab/2125?Ntk=Products&Ntt=2125), b-actin (#4970, Cell Signaling, https://www.cellsignal.com/products/primary-antibodies/b-actin-13e5-rabbit-mab/4970?Ntk=Products&Ntt=4970), CD45 (#REA747, Miltenyi Biotec, https://www.miltenyibiotec.com/US-en/products/cd45-antibody-anti-human-reafinity-rea747.html#vioblue:100-tests-in-200-ul), CD31 (#REA730, Miltenyi Biotec, https://www.miltenyibiotec.com/US-en/products/cd31-antibody-anti-human-reafinity-rea730.html#apc:100-tests-in-200-ul), VEGFR-2 (#REA1046, Miltenyi Biotec, https://www.miltenyibiotec.com/US-en/products/cd309-vegfr-2-antibody-anti-human-reafinity-rea1046.html#apc-vio-770:30-tests-in-60-ul), CD62P (#REA389, Miltenyi Biotec,

https://www.miltenyibiotec.com/US-en/products/cd62p-antibody-anti-human-reafinity-rea389.html#pe-vio-770:100-tests-in-200-ul), and CD62P (#550888, BD Biosciences, https://www.bdbiosciences.com/us/applications/research/t-cell-immunology/regulatory-t-cells/surface-markers/human/apc-mouse-anti-human-cd62p-ak-4-also-known-as-ak4-or-ak-4/p/550888) have been validated by the manufacturer and are extensively used in the scientific community.

Eukaryotic cell lines

Policy information about cell lines

Cell line source(s)	The MM cell lines, MM.1S and H929, were purchased from American Type Culture Collection (ATCC, Rockville, MD). OPM-2 and green fluorescent protein-labeled and luciferase-transfected MM.1S (MM.1S-GFP-Luc) were a kind gift from Dr. Irene Ghobrial (Dana-Farber Cancer Institute, Harvard Medical School, Boston, MA).
Authentication	The cell lines were not authenticated in our lab.
Mycoplasma contamination	All cell lines tested negative for mycoplasma.
Commonly misidentified lines (See ICLAC register)	There were no commonly misidentified cell lines used in this study.

Animals and other organisms

Policy information about studies involving animals; ARRIVE guidelines recommended for reporting animal research

Laboratory animals	All animal work presented in this study was approved by the Institutional Animal Care and Use Committee at Washington University School of Medicine in St. Louis. NCG (NOD-Prkdcem26Cd52Il2rgem26Cd22/NjuCrl) male 50-56 day-old mice (Charles River, Wilmington, MA) were used in this study. All animals were housed on a light-dark cycle of 12 hours daily in a temperature- and humidity-controlled facility at Washington University School of Medicine.
Wild animals	No wild animals were used in this study.
Field-collected samples	No field-collected samples were used in this study.
Ethics oversight	All animal work presented in this study has been approved by the Institutional Animal Care and Use Committee at Washington University School of Medicine in St. Louis.

Note that full information on the approval of the study protocol must also be provided in the manuscript.

Human research participants

Policy information about studies involving human research participants

Population characteristics	Patient samples used in this study were either healthy or had multiple myeloma. Of those who had multiple myeloma, these patients were either newly diagnosed or relapsed. The treatments of the relapsed patients were random. Ages of the subjects ranged from 18 - 90 years old. The genders of the subjects were both male and female.
Recruitment	Patient samples used were selected at random. Our clinician collaborators selected the samples at random and gave them to our lab for further analysis therefore no bias was introduced in the study.
Ethics oversight	Washington University ethics committee has approved this study, and all patients provided written informed consent (IRB #201102270). This study was performed in accordance with the ethical standards detailed in the Declaration of Helsinki.

Note that full information on the approval of the study protocol must also be provided in the manuscript.

Flow Cytometry

Plots

Confirm that:

- [x] The axis labels state the marker and fluorochrome used (e.g. CD4-FITC).
- [x] The axis scales are clearly visible. Include numbers along axes only for bottom left plot of group (a 'group' is an analysis of identical markers).
- [x] All plots are contour plots with outliers or pseudocolor plots.
- [x] A numerical value for number of cells or percentage (with statistics) is provided.

Methodology

Sample preparation	For flow cytometry, the cells were taken from the 96-well plate, washed with 1X PBS, and spun down at 1000 rpm for five minutes. Then the supernatant was aspirated, and the sample was resuspended in fresh 1X PBS. The samples were incubated in the antibodies of interest for one hour and subsequently spun down at 1000 rpm for five minutes. Following spinning, the samples were resuspended in 1X PBS and ran through the flow cytometer for fluorescence of incubated antibody.
Instrument	MACSQuant Analyzer
Software	MACSQuantify and FlowJo were used during data collection and analysis, respectively.
Cell population abundance	Abundance and purity in the desired cell populations were assessed by flow cytometry analysis.
Gating strategy	The preliminary FSC/SSC plots of the starting cell population were gated to only include the living cells. Then a clear separation between the positive and negative cell populations in the channel of interest was defined to distinguish the fluorescent from the non-fluorescent cells.

☒ Tick this box to confirm that a figure exemplifying the gating strategy is provided in the Supplementary Information.
